# Supplementary material for: Prevalence and correlates of symptoms of post-traumatic stress disorder among Chinese healthcare workers exposed to physical violence: a cross-sectional study
Source: BMJ Open. 2017 Aug 1;7(7):e016810. doi: 10.1136/bmjopen-2017-016810 (PMC5642665; doi:10.1136/bmjopen-2017-016810)
Supplement: Author's manuscript [file bmjopen-2017-016810.draft_revisions.pdf]

BMJ Open is committed to open peer review. As part of this commitment we make the peer review history of every article we publish publicly available.

When an article is published we post the peer reviewers' comments and the authors' responses online. We also post the versions of the paper that were used during peer review. These are the versions that the peer review comments apply to.

The versions of the paper that follow are the versions that were submitted during the peer review process. They are not the versions of record or the final published versions. They should not be cited or distributed as the published version of this manuscript.

BMJ Open is an open access journal and the full, final, typeset and author-corrected version of record of the manuscript is available on our site with no access controls, subscription charges or pay-per-view fees (http://bmjopen.bmj.com).

If you have any questions on BMJ Open's open peer review process please email editorial.bmjopen@bmj.com

BMJ Open

Prevalence and correlates of symptoms of post-traumatic stress disorder among Chinese healthcare workers exposed to physical violence : a cross-sectional study

Journal:	*BMJ Open*
Manuscript ID	bmjopen-2017-016810
Article Type:	Research
Date Submitted by the Author:	12-Mar-2017
Complete List of Authors:	Shi, Lei<br>Wang, Lingling ; Chinese Hospital Association, Department of Autonomous Protection<br>Jia, Xiaoli<br>Li, Zhe<br>Mu, Huitong<br>Liu, Xin<br>Peng, Boshi<br>Li, Anqi<br>Fan, Lihua; Harbin Medical University, Department of Health Management , School of Public Health
**Primary Subject Heading**:	Health policy
Secondary Subject Heading:	Health policy, Health services research, Mental health
Keywords:	Posttraumatic stress disorder symptoms (PTSD), social support, coping styles, MENTAL HEALTH, physical violence, personality

SCHOLARONE™
Manuscripts

Prevalence and correlates of symptoms of post-traumatic stress disorder among Chinese healthcare workers exposed to physical violence : a cross-sectional study

Lei Shi,[1] Lingling Wang,[2] Xiaoli Jia,[2] Zhe Li,[1] Huitong Mu,[1] Xin Liu,[1] Boshi Peng,[3] Anqi Li,[1] Lihua Fan[1]

Correspondence to: Lihua Fan, School of Public Health, Harbin Medical University, 157 Baojian Road, Nangang District, Harbin 150081, China; E-mail: lihuafan@126.com

[1]Department of Health Management, School of Public Health, Harbin Medical University, Harbin, Heilongjiang, China

[2]Department of Autonomous Protection, Chinese Hospital Association, Beijing, China

[3] Department of Scientific Research, The Second Affiliated Hospital of Harbin Medical University, Harbin, Heilongjiang, China

For peer review only - http://bmjopen.bmj.com/site/about/guidelines.xhtml

Abstract

Objectives

Post-traumatic stress disorder (PTSD) is a common psychological maladjustment among healthcare workers after undergoing a traumatic event. Our aim was to measure the prevalence of PTSD symptoms, and to explore the associations of demographic characteristics, social support, personality traits, and coping styles with PTSD symptoms among Chinese healthcare workers exposed to physical violence.

Methods

A cross-sectional study was conducted using standard questionnaires: the Workplace Violence Scale, the Post-traumatic Stress Disorder Checklist-Civilian Version, the Social Support Rating Scale (SSRS), the Revised Eysenck Personality Questionnaire-Short Scale (EPQ-RSC) and Trait Coping Style Questionnaire (TCSQ).We employed a convenient sampling method to collect data from March 2015 to September 2016.Atotal of 2706 participants from 39 public hospitals located in Heilongjiang, Hebei, and Beijing Provinces of China(effective response rate = 84.25%).This study was only about physical violence, 368 participants were eligible for the study. Pearson's correlations were used to examine correlations among continuous variables. Hierarchical regression analysis were used to examine the associations of the demographic characteristics and scores on the SSRS, EPQ-RSC, and TCSQ with PTSD symptoms.

Results

Overall, the prevalence of physical violence in the previous 12 months was 13.60%.The prevalence of PTSD symptoms was 28.0%. Most of the participants (47.0%) did not appear to be have PTSD symptoms after experiencing physical violence. The healthcare workers adopted negative coping with

For peer review only - http://bmjopen.bmj.com/site/about/guidelines.xhtml

physical violence was positively associated with the development of PTSD symptoms ($\beta = 0.179$, $P < 0.01$). As expected, social support was negatively associated with PTSD symptoms ($\beta = -0.129$, $P < 0.05$) and was a protective factor. In women, positive coping of TCSQ was significantly associated with PTSD symptoms ($\beta = -0.229$, $P < 0.01$). However, the effect of positive coping was not significant in men.

Conclusions

The prevalence of PTSD symptoms among healthcare workers who experienced physical violence was high. The positive effects of social support on PTSD symptoms suggest that organizational and familial support has practical implications for interventions to promote psychological health. The healthcare workers' personalities and coping styles also influenced development of the PTSD symptoms. Therefore, it is imperative to adopt correct coping styles after experiencing a traumatic events.

Strengths and limitations of this study

- In China, there were relatively few studies on PTSD symptoms after healthcare workers exposed to physical violence.
- We assessed the prevalence of PTSD symptoms, and to explore the correlates of PTSD symptoms among Chinese healthcare workers exposed to physical violence.
- Our study was only conducted at 39 public hospitals of three provinces, and large sample size research could contribute to the generalization of our findings.
- The retrospective approach to self-reported PTSD symptoms used by respondents may cause recall and report bias.

For peer review only - http://bmjopen.bmj.com/site/about/guidelines.xhtml

BACKGROUND

Post-traumatic stress disorder (PTSD) is generally recognized as a psychological state of imbalance, characterized by a series of chronic emotional reactions to a traumatic event, including re-experiencing, avoidance and heightened arousal.[1-3] A substantial number of studies indicate that almost all people exhibit intrusive and repetitious symptoms after exposure to excessive stress,[4] however, only a small percentage develop avoidance and hyper-arousal symptoms. Therefore, most individuals showing PTSD symptoms after exposure to a traumatic event recover within weeks or months. However, 10%–25% might develop chronic PTSD that lasts for several months, years, or even a lifetime.[5] PTSD originated from reports of the war trauma, and then was applied gradually to a variety of man-made and natural disasters.[6] Scholars have reported that the incidence of PTSD among male and female Vietnam veterans in the USA is 15.2% and 8.5%, respectively.[7] There are differences in the incidence rates of PTSD and for various types of trauma in China. For instance, PTSD was reported to be 8.65% among soldiers assigned to military vehicles at high altitudes, 33.89% in flood disaster survivors, 18.8% in earthquake survivors, 41% in traffic accident survivors and 78.6% in survivors after a serious explosion.[8] Therefore, most of the Chinese studies on PTSD have focused on wars, traffic accidents, and natural disasters.[9-11]

Previous studies have found that the death of a child, medical emergencies, accidental trauma, workplace violence, suicide and issues related to hospital management are ranked as the top six trauma events seen in hospitals.[12] Physical violence not only leads to direct economic loss, death and physical injury, but also cause long-term adverse psychological consequences.[13-16] Several studies have estimated the prevalence of PTSD symptoms among emergency department (ED) staff to range from 10% to 25%, which might be attributed to differences in the studies' sample characteristics, designs,

For peer review only

For peer review only - http://bmjopen.bmj.com/site/about/guidelines.xhtml

definitions, and diagnostic tools for PTSD due to their varied cultural backgrounds.[17-19] There are also reports of the occurrence of PTSD among Chinese nurses working in the ED, intensive care unit, and operating room. However, the number of research studies on PTSD among healthcare workers has been relatively few in China.

Demographic variables (e.g., age, gender, and educational level), and psychological and social variables (e.g., personality, attribution style, and social support) have been found to be significantly correlated with violence-related PTSD symptoms.[20-23] Social support has been found to be an effective emotional regulator under conditions of traumatic stress.[24] Furthermore, coping styles and personality characteristics have also been identified as factors influencing the development of PTSD symptoms.[25-26]

In this study, we aimed to assess the prevalence of PTSD symptoms, and to explore the associations of demographic characteristics, social support, personality characteristics, and coping styles with PTSD symptoms among Chinese healthcare workers exposed to physical violence.

METHODS

Participants and procedure

Between March 2015 through September 2016, a cross-sectional study was conducted with a sample of healthcare workers employed by 39 public hospitals located in Heilongjiang, Hebei, and Beijing Provinces of China. The 39 public hospitals that served as the research settings were chosen with the use of a convenience sampling method. All investigators were trained by a unified written survey before they began to collect data. Qualified investigators were appointed to collect data. We obtained permission from the managers, medical dispute resolution and human resources departments of the

For peer review only - http://bmjopen.bmj.com/site/about/guidelines.xhtml

hospitals. The investigators carried out face-to-face survey by using an anonymous, self-administered questionnaire. We purposely selected 3 public hospitals of Harbin (The First Affiliated Hospital of Harbin Medical University, The Second Affiliated Hospital of Harbin Medical University, The Fourth Affiliated Hospital of Harbin Medical University) as a site for our pilot study site before the formal investigation. A total of 150 questionnaires were distributed and recovered. A total of 3,212 healthcare workers (physicians, nurses and medical technician) were investigated using a convenience sampling method in the formal investigation. The researchers and hospital coordinators distributed and collected questionnaires that were completed by the healthcare workers immediately. We eliminated the questionnaires that existed missing data or quality problems. A total of 2,706 valid questionnaires were returned, and the effective response rate was 84.25%.This study was only about physical violence, so the 368 healthcare workers are suitable for research.

The inclusion criteria for participants in this study were as follows: (1) being at least one year of work experience; (2) volunteered to participate; (3) would not affect their work; and (4) experienced physical violence in the previous 12 months. Excluded from this study were individuals who (1) had received any psychological treatment after experienced physical violence, and (2) experienced other traumatic events.

QUESTIONNAIRE

Demographic characteristics

Demographic characteristics including gender, age, marital status, professional title, occupation, and work experience, etc.

Workplace Violence Scale

For peer review only - http://bmjopen.bmj.com/site/about/guidelines.xhtml

The Workplace Violence Scale developed by the International Labour Office, International Council of Nurses, World Health Organization, and Public Services International Joint Programme on Workplace Violence in the Health Sector in 2003 and revised Survey of Violence Experienced by Staff (SOVES-G-R) was used to measure workplace violence.[27-28] We obtained permission to use this scale. The scale is divided into 2 dimensions and has 9 items. Each item is scored on a 4-point scale to reflect respondents' frequency of exposure to violence (0 = 0 times, 1 = 1 time, 2 = 2–3 times,3 = ≥4 times). The total possible score ranges from 0 to 27, with a higher total score indicating a higher frequency of exposure to WPV. In the present study, Cronbach's α for the Workplace Violence Scale was 0.86.

PTSD

The PTSD Checklist-Civilian Version (PCL-C) was used to measure PTSD symptoms of the healthcare workers.[29] It consists of 17 self-report items and three dimensions, namely, re-experiencing (items 1–5), avoidance/numbing (items 6–12) and hyper-arousal (items 13–17). The three dimensions correspond to the DSM-IV symptoms criteria.[2] The options for each item on the PCL-C are rated from 1 (not at all) to 5 (extremely) based on the extent to which the respondent has been troubled by specific symptoms in the past month. The total possible score is calculated by adding the scores for all items, and it ranges from 17 to 85, with a higher score indicating a higher risk for PTSD symptoms. A total score ≥ 50 is indicative of PTSD symptoms.[29] In this study, the traumatic event in the original PCL-C was replaced by the physical violence. The reliability and validity of this instrument have been shown to be high in a wide range of Chinese populations.[30-32] The present study revealed that Cronbach's α for the PCL-C was 0.934, and for the three sub-scales it was 0.872 (re-experiencing), 0.921(avoidance/numbing), and 0.926 (hyper-arousal).

Eysenck Personality Questionnaire

For peer review only - http://bmjopen.bmj.com/site/about/guidelines.xhtml

Personality traits were measured using the Eysenck Personality Questionnaire-Revised Short Scale for Chinese (EPQ-RSC). The EPQ-RSC consists of 48 items, categorized into 4 subscales reflecting personality traits: Extraversion, Neuroticism, Psychoticism, and Lie. Each item is scored on a dichotomous scale (1=Yes, 0=No) to measure personality characteristics. The scores of the positively and negatively worded items are summed in accordance with each personality trait. Early studies have found the EPQ-RSC to have high reliability and validity as a measure of personality traits in China.[33-34] The total score for the Extraversion subscale indicates introversion when it is less than 43.3, middle when it is from 43.3 to 56.7 and extraversion when it is greater than 56.7. For the Psychoticism subscale, tough-minded is defined when the total score is greater than 56.7; middle is defined when the total score is from 43.3 to 56.7, and mild is defined when the total score is less than 43.3. For the Neuroticism subscale, a total score of less than 43.3 defines emotional stability, while a total score from 43.3 to 56.7 defines middle, and a total score greater than 56.7 defines emotional instability.[33] For the Lie subscale, a total score for 60 or greater may indicates information provided by respondents is unreliable.[33] In this study, Cronbach'sα for the EPQ-RSC was 0.903. The internal consistency coefficients were 0.854, 0.756, 0.791, and 0.762, for the Extraversion, Neuroticism, Psychoticism, and Liesubscales, respectively.

Trait Coping Style Questionnaire

The Trait Coping Style Questionnaire (TCSQ) was used to assess participants' coping styles of life events in this study. The TCSQ consists of 20 items, including 10 items of positive coping (items 1, 3, 5, 8, 9, 11, 14, 15, 18, 20) and negative coping (items 2, 4, 6, 7, 10, 12, 13, 16, 17, 19), respectively. Each item is rated on a 5-point Likert scale. The total possible score of positive and negative coping is calculated by adding the scores for all items. Previous studies have found the TCSQ to have high

For peer review only - http://bmjopen.bmj.com/site/about/guidelines.xhtml

reliability and validity as a measure of coping style in China.[35-36] In this study, Cronbach's α for the total scale was 0.845, and the internal consistency coefficients of the subscales were α=0.823 (positive coping), and α=0.863 (negative coping).

Social Support Rating Scale

Social support was evaluated using the Chinese version of the Social Support Rating Scale (SSRS),[37-38] which is a short measure of the social support individuals have received. This 10-item scale is divided into 3 dimensions: subjective support (items 1, 3, 4, 5), objective support (items 2, 6, 7) and the availability of support (items 8, 9, 10). Social support level is defined as low when the total score is from 12 to 44, medium when the total score is from 45 to 54, and high when the total score is greater than 55.[39] The present study revealed that the Cronbach'a α for the SSRS was 0.865, and for the three subscales it was 0.884 (subjective support), 0.911 (objective support), and 0.875 (the availability of support).

DATA ANALYSIS

EpiData version 3.1 was used to establish the study's database. Data were double entered after carefully checking and eliminating data that did not qualify for the statistical. IBM SPSS Version 19.0 and Excel were used for the data analysis. The normal distributions of the continuous variables were verified using P-P plots and K-S tests. Descriptive statistics, including numbers (n), percentages (%), means, and standard deviations (SD)were calculated for the demographic variables.. We used one-way analysis of variance (ANOVA)or independent sample t-tests to compare the group differences of measures of the continuous variables. The chi-square (χ^2) test was used to compare differences in categorical variables. Pearson's correlations were used to examine correlations among continuous

For peer review only - http://bmjopen.bmj.com/site/about/guidelines.xhtml

variables. Hierarchical regression analysis was used to examine the associations of the demographic characteristics and scores on the SSRS, EPQ-RSC, and TCSQ with PTSD symptoms. Data including F value, R^2, R^2-changes (ΔR^2), standardized regression coefficient (β) and P-value for each step in the regression model were reported. All the study variables were tested for multi-colinearity. A P-value<0.05 was considered to be statistically significance.

ETHICAL CONSIDERATIONS

Ethical approval to undertake this study was granted by the Research Ethics Committee of Harbin Medical University, and informed consent was obtained from each hospital and healthcare worker involved in the investigation. All of the participants gave the informed consent before the survey; they were assured that their personal information would be kept confidential.

RESULTS

Sample demographics

The demographic characteristics of the participants are shown in Table 1. Of the 368 participants, 59.8% were women, 51.3% received an undergraduate education, and 73.9% were married. The prevalence of physical violence in the previous 12 months was 13.60%.

Table 1. Characteristics of participants in relation to PTSD symptoms. (N=368).

Variables		n	%	PTSD symptoms		F/t	*P*
				Mean	SD		
Gender	Male	148	40.2	44.03	16.19	3.537	0.000
	Female	220	59.8	38.30	13.71		
Age group	≤30	133	36.1	38.09	13.36	2.946	0.054
	31-50	216	58.7	42.01	15.56		
	≥51	19	5.2	42.10	17.80		
Education status	Junior college or below	118	32.1	38.14	13.54	2.592	0.076

For peer review only - http://bmjopen.bmj.com/site/about/guidelines.xhtml

	Undergraduate	189	51.3	42.13	15.46		
	Graduate	61	16.6	40.64	15.85		
Marital status	Married	272	73.9	41.51	15.75	2.195	0.029
	Single/ divorced/ widowed	96	26.1	38.03	12.38		
Occupation	Physician	175	47.6	42.97	15.37	4.379	0.013
	Nurse	180	48.9	38.29	13.82		
	Medical Technician	13	3.5	40.69	21.24		
Technical title	Primary	145	39.4	39.56	13.04	0.576	0.562
	Intermediate	126	34.2	41.32	16.21		
	Senior	97	26.4	41.23	16.16		
Department	Emergency Department	68	18.5	41.46	16.08	0.722	0.607
	Internal Medicine	76	20.7	38.45	15.07		
	Surgery	123	33.4	41.53	13.91		
	Obstetrics and Gynecology	19	5.2	41.63	16.52		
	Pediatrics	27	7.3	37.63	10.37		
	Other	55	14.9	41.55	17.28		
Years of experience	≤4	101	27.4	37.19	13.25	2.158	0.063
	5-10	120	32.6	42.13	14.52		
	11-20	87	23.7	41.90	16.80		
	≥21	60	16.3	41.42	15.46		
Social support	Low	224	60.9	42.41	15.06	5.904	0.003
	Medium	130	35.3	38.52	14.40		
	High	14	3.8	31.00	14.53		
Extraversion	Introversion	102	27.7	39.45	13.35	1.278	0.280
	Middle	164	44.6	41.99	16.04		
	Extraversion	102	27.7	39.51	14.80		
Psychoticism	Mild	68	18.5	42.22	16.87	0.998	0.370
	Middle	213	57.9	40.79	15.24		
	Tough-minded	87	23.6	38.86	12.69		
Neuroticism	Emotional instability	100	27.2	40.33	13.80	0.530	0.589
	Middle	153	41.6	41.50	16.72		
	Emotional stability	115	31.2	39.63	13.60		

The prevalence of PTSD

The PTSD symptoms based on participants' PCL-C scores are summarized in Table 2. According to their scores on the PCL-C, 187 participants (28.0%) showed PTSD symptoms. The participants (21.2%) were considered to be at risk for later developing PTSD.

According to the DSM IV-TR criteria for PTSD,[2] most of the participants did not appear to be have PTSD symptoms. The criterion for PTSD that was the most frequently observed in the physical

For peer review only - http://bmjopen.bmj.com/site/about/guidelines.xhtml

For peer review only

violence group was re-experiencing. The criterion for PTSD that was the least frequently happening criterion for PTSD observed in the physical violence group was avoidance.

Table2. Sample description and prevalence of PTSD symptoms.

PTSD symptoms	Physical violence	
	n	%
**PTSD symptoms based on PCL-C scores**		
No obvious PTSD symptoms (17-37)	187	50.8
Criteria met for potential risk of PTSD symptoms(38-49)	78	21.2
Criteria met for PTSD symptoms(50-85)	103	28.0
**PTSD symptoms based on PTSD criterion***		
No criterion manifestation	173	47.0
Re-experiencing (Criterion B)	166	45.1
Avoidance (Criterion C)	129	35.1
Hyper-arousal (Criterion D)	139	37.8

Note. PTSD = posttraumatic stress disorder; PCL-C = PTSD Checklist Version.

* Participants may have more than one criteria.

The correlations of the EPQ-RSC, TCSQ and SSRS scores with PTSD symptoms

Table 3 shows the correlations among the participants' PTSD symptoms and scores on the EPQ-RSC, TCSQ, and SSRS. The mean score for PTSD symptoms on the PCL-C was 40.60 (SD = 15.00).The mean score for the SSRS was 41.73 (SD = 8.44). The mean score for positive coping and negative coping was 30.05 (SD = 7.22) and 26.92 (SD = 7.33). As expected, the level of PTSD symptoms was negatively correlated with their scores on the SSRS(r=-0.188, P= 0.000).and positive coping of TCSQ (r=-0.164, P= 0.002), respectively. The level of PTSD symptoms was positively correlated with participants' scores on the to negative coping of TCSQ (r=0.188, P= 0.000).

Table 3. Pearson correlations among PTSD symptoms, EPQ-RSC, TCSQ and SSRS.

Variables	1	2	3	4	5	6
1.PTSD symptoms	-					
2. SSRS	-0.188**	-				
3. Positive coping of TCSQ	-0.164**	0.101	-			
4. Negative coping of TCSQ	0.188**	-0.310**	0.123*	-		
5.Extraversion	-0.007	-0.045	-0.036	0.015	-	
6. Psychoticism	0.057	0.017	0.023	0.043	-0.023	-
7. Neuroticism	0.027	0.025	0.007	0.035	-0.091	0.199**

*P<0.05, **P<0.01

For peer review only - http://bmjopen.bmj.com/site/about/guidelines.xhtml

Hierarchical regression analysis of related factors of PTSD symptoms

The results of the hierarchical regression analysis of the variables are presented in Table 4. Variables that had a statistically significant association with PTSD were used as control variables. Gender had a significant effect on PTSD symptoms in the model (Block 1).As shown in Block 2, social support was negatively associated with PTSD symptoms (β = -0.211, P = 0.018). On the other hand, negative coping of TCSQ was positively associated with PTSD symptoms in the regression model (β = 0.176, P = 0.001). As shown in Block 3, Psychoticism of EPQ-RSC was positively associated with PTSD symptoms in the regression model(β = 0.054, P = 0.014). Further, gender had a significant effect on PTSD symptoms, and men were more vulnerable to PTSD symptoms than women (Table 1). Therefore, we explored the potential correlates of PTSD symptoms in men and women, respectively (Table 5). As shown in Block 2, in women, positive coping of TCSQ was significantly associated with PTSD symptoms (β = -0.229, P = 0.001). However, the effect of positive coping of TCSQ was not significant in men.

Table 4. Hierarchical regression for exploring the positive correlates of PTSD symptoms.

Variables	Block 1 (β)	Block 2 (β)	Block 3 (β)
Gender	0.153**	0.132*	0.137*
Marital status	-0.059	-0.117*	-0.088
Occupation	-0.048	-0.044	-0.072
SSRS		-0.211**	-0.129*
Positive coping of TCSQ		-0.182**	-0.181**
Negative coping of TCSQ		0.176**	0.179**
Extraversion			-0.023
Psychoticism			0.154*
Neuroticism			0.022*
F	5.189**	12.533**	16.263**
R^2	0.041	0.131	0.236
ΔR^2	0.041	0.090**	0.105**

*P<0.05, **P<0.01

For peer review only - http://bmjopen.bmj.com/site/about/guidelines.xhtml

Table 5. Hierarchical regression for exploring the correlates of PTSD symptoms in men and women, respectively.

	Variables	Mean (SD)	Block 1 (β)	Block 2 (β)
Male				
n = 148	SSRS	41.25 (9.32)	-0.158	-0.159
	Positive coping of TCSQ	30.75 (7.28)	-0.152	-0.158
	Negative coping of TCSQ	27.21 (6.61)		0.208*
	Extraversion	49.18 (10.04)		0.070
	Psychoticism	50.02 (8.62)		-0.135
	Neuroticism	49.99 (10.59)		-0.032
	F		5.501	9.523
	R^2		0.103	0.231
	ΔR^2		0.103	0.128
Female				
n = 220	SSRS	42.05 (7.79)	-0.059	-0.064
	Positive coping of TCSQ	29.58 (7.16)	-0.229**	-0.229**
	Negative coping of TCSQ	26.72 (7.78)	0.167*	0.168*
	Extraversion	50.03 (10.54)		-0.094
	Psychoticism	50.14 (10.56)		0.000
	Neuroticism	50.12 (10.20)		-0.003
	F		6.726	10.681
	R^2		0.085	0.194
	ΔR^2		0.085	0.109*

$^{*}P<0.05$, $^{**}P<0.01$

DISCUSSION

In this cross-sectional hospital-based study of healthcare workers exposed to physical violence, we assessed the prevalence and correlates of PTSD symptoms. Our study found that the prevalence of physical violence was about 13.6% in the previous year. PTSD symptoms were reported by 28.0% of the healthcare workers based on the scoring instructions of the PCL-C (i.e., 28.0% scored 50 points and above). We selected the PCL-C score of 50 and above as the standard cut-off due to the influence of traditional Chinese culture on the frequency of healthcare workers' encounters with traumatic events, and the DSM IV-TR criteria for PTSD.[2] Previous studies have provided valuable information regarding the prevalence of PTSD symptoms.[17 40-41] The prevalence of PTSD symptoms in our sample was higher than that of general population (8%) in the USA.[42] This finding might be attributed to the fact

For peer review only - http://bmjopen.bmj.com/site/about/guidelines.xhtml

that the general population’s exposure to traumatic events is less than that of healthcare workers. Similarly, intensive care unit nurses experience traumatic events more often than other healthcare workers do.[18]

Our study found that 21.2% of the healthcare workers might develop PTSD symptoms and 28.0% of the healthcare workers appeared PTSD symptoms after experiencing physical violence. This phenomenon revealed that physical violence had a strong influence on the mental health of the healthcare workers. Approximately 53.0% (195/368) of the participants reported having at least one PTSD criterion. The PTSD symptoms that was the most commonly observed was re-experiencing, followed by hyper-arousal, and then avoidance. A previous study also reported that the healthcare workers of emergency department were the direct victims of workplace violence because they reported re-experiencing the violent event, followed by hyper-arousal, and avoidance.[17] This finding might reflect the normal stress response of healthcare workers and support the notion that some healthcare workers might benefit from relaxation training and psychological interventions by professionals. The symptoms of hyper-arousal (37.8%) and re-experiencing (45.1%) were reported by participants after experiencing physical violence. Previous studies revealed that re-experiencing and hyper-arousal the violence incident was significantly and negatively associated with emergency department workers’ ability to accomplish their work.[17 43] Approximately 47.0% of the sample reported no symptoms manifestation after experiencing physical violence. This phenomenon might be related to hospital culture, which requires healthcare workers to be able to shift their focus quickly and constantly. Healthcare workers who escaped slight injury during an episode of physical violence had to shift their rapidly focus to another patient after the event.

As shown in results of the Pearson’s correlations and hierarchical regression analysis, social

For peer review only - http://bmjopen.bmj.com/site/about/guidelines.xhtml

support had a significant negative association with PTSD symptoms, and this finding is consistent with other research.[10 24 32 36] A supportive environment can help individuals cope with all kinds of stressful events, and serve as a buffer against their negative health effects. Social support might be especially important for healthcare workers; because of the unique aspects of their jobs, they need support from patients, colleagues, friends and relatives. In addition, a significant effect of coping styles on PTSD symptoms was found in the present study. This result indicated that when healthcare workers encountered a traumatic event, a negative coping was more likely to increase their proneness to developing PTSD symptoms. In contrast, positive coping was beneficial to prevent or alleviate PTSD symptoms. Surprisingly, introverted and stable personalities were negatively associated with PTSD symptoms. One possible reason is that a balanced disposition i.e., one that is intermediate between extroversion and introversion is more conducive to the psychological health of healthcare workers. Meanwhile, emotional instability and characteristics of an tough-minded personality were risk factors for developing PTSD symptoms. This personality is not conducive to communication with people, thereby increasing the person's vulnerability.[44]

An important finding of the present study was revealed in the univariate analyses. We found that the men exposed to traumatic events were more likely to exhibit PTSD symptoms than the women were. This result was different from the findings of earlier studies that women are more likely to develop PTSDsymptoms.[6 17 19] This might be attributed to gender differences in coping styles and social support. This phenomenon also may be attributable to the fact that the severity of injury after experiencing physical violence in men was higher than women. Women were likely to get more social support than men after experiencing physical violence, and it may be that women were often regarded as vulnerable groups. We also found that men were more likely to respond negatively to traumatic

For peer review only - http://bmjopen.bmj.com/site/about/guidelines.xhtml

events. These findings suggest that social support, coping styles, whether a person was exposed to physical violence, emotional instability, and anxious personality is closely related to PTSD symptoms. Therefore, it is necessary to implement interventions, For example, specialized or routine psychological interventions should be developed by a clinical psychologist, and hospitals could provide violence-related training for healthcare workers and provide psychological support or a "debriefing room."[45] These interventions should help to in reduce PTSD symptoms.

There are several limitations of the present study. First, we used the PCL-C to assess PTSD symptoms rather than a standard clinical diagnostic method. Consequently, the prevalence of PTSD might be overestimated. Second, the findings need to be confirmed in a longitudinal study. Finally, our results are specific to Chinese healthcare workers exposed to physical violence in the past 12-month. Thus, the inclusion of additional careers and a larger sample size should contribute to the validity the results of future studies.

CONCLUSIONS

Healthcare workers who have experienced physical violence are more likely to develop PTSD symptoms. The positive effects of social support on PTSD symptoms suggest that organizational and familial support have practical implications for psychological interventions to promote health. Furthermore, the personalities and coping styles of the healthcare workers have influenced the development of PTSD symptoms. It is imperative to keep positive coping and get social support after experiencing traumatic events.

Acknowledgments

For peer review only - http://bmjopen.bmj.com/site/about/guidelines.xhtml

The authors thank all the healthcare workers, managers and Chinese Hospital Association for their assistance and support for this project.

Author Contributions

LS and LF designed the study. LS, LW, XJ, BP and LF collected data. ZL, LW, XJ, HM, XL and AL analysed the data. LS and LF drafted the manuscript. LS, ZL and LF revised the manuscript.

Funding

This study was funded by the National Natural Science Foundation of China, grant number 71473063.

Competing interests None declared.

Data sharing statement No additional data are available.

REFERENCES

1. Guo W, Xue JM, Shao D, *et al.* Effect of the interplay between trauma severity and trait neuroticism on posttraumatic stress disorder symptoms among adolescents exposed to a pipeline explosion. *PLoS One* 2015;10(3):e0120493.

2. Association AP. Diagnostic and Statistical Manual of Mental Disorders fourth edition, Text revision (DSM-IV TR). 2000.

3. Andreasen NC. What is post-traumatic stress disorder? *Dialogues Clin Neurosci* 2011;13:240–243.

4. Van Der Kolk BA.The psychobiology and psychopharmacology of PTSD. *Hum Psychopharmacol* 2001;16(S1):S49 – S64.

5. Shalev AY. Post-traumatic stress disorder. Disorder takes away human dignity and character. *BMJ* 2001;322(7297):1301–1304.

For peer review only - http://bmjopen.bmj.com/site/about/guidelines.xhtml

6. Hu BS, Liang YX.Post-traumatic stress disorder. *Journal of Environmental Hygiene* 1997;(5): 266-269.

7. Klein S, Alexander DA. Epidemiology and presentation of post-traumatic disorders. *Psychiatry-interpersonal & Biological Processes* 2009;8(8):282-287.

8. Wang YL, Xie W, Yang ZH, *et al.* Research progress of posttraumatic stress disorder in China. *Chinese Journal of Applied Psychology* 2005;11(2):176-180.

9. Brewin CR, Andrews B, Valentine JD. Meta-analysis of risk factors for posttraumatic stress disorder in trauma exposed adults. *J Consult Clin Psycho* 2000;68(5):748–766.

10. Fan F, Zhang Y, Yang Y,*et al.* Symptoms of posttraumatic stress disorder, depression, and anxiety among adolescents following the 2008 Wenchuan earthquake in China. *J Trauma Stress* 2011;24(1): 44–53.

11. Boe HJ, Holgersen KH, Holen A. Mental health outcomes and predictors of chronic disorders after the North Sea oil rig disaster: 27-year longitudinal follow-up study. *J Nerv Ment Dis* 2011;199(1):49–54.

12. van der PE, Kleber RJ. Acute and chronic job stressors among ambulance personnel: predictors of health symptoms. *Occup Environ Med* 2003;60:i40-6.

13. Hashmi S, Petraro P, Rizzo T, *et al.* Symptoms of anxiety, depression, and posttraumatic stress among survivors of the 2005 Pakistani earthquake. *Disaster Medicine and Public Health Preparedness*2011;5(4):293.

14. van Griensven F, Chakkraband ML, Thienkrua W, *et al.* Mental health problems among adults in tsunami-affected areas in southern Thailand. *JAMA*2006;296(5):537-548.

15. Ahmad M, Al-Rimawi R, Masadeh A, *et al.* Workplace Violence by Patients and their Families

For peer review only - http://bmjopen.bmj.com/site/about/guidelines.xhtml

against Nurses: Literature Review. *INT J NURS TERMIN CL* 2015;2(4):46-55.

16. Ramacciati N, Ceccagnoli A, Addey B. Violence against nurses in the triage area: An Italian qualitative study. *International Emergency Nursing*, 2015;23(4):274-280.

17. Laposa JM, Alden LE. Posttraumatic stress disorder in the emergency room: exploration of a cognitive model. *Behav Res Ther* 2003;41(1):49-65.

18. Mills LD, Mills TJ. Symptoms of post-traumatic stress disorder among emergency medicine residents. *J Emerg Med* 2005;28(1):1–4.

19. Friedman, MJ, Resick, PA, Bryant, R.A, *et al.* Considering PTSD for DSM-5. *Depression and Anxiety* 2011;28(9):750-769.

20. Kornblith AB, Herndon JE, Weiss RB, *et al.* Long-term adjustment of survivors of early-stage breast carcinoma, 20 years after adjuvant chemotherapy.*Cancer*2003;98(4):679–689.

21. Rourke MT, Hobbie WL, Schwartz L, *et al.* Posttrauamatic stress disorder (PTSD) in young adult survivors of childhood cancer. *Pediatr Blood Cancer* 2007; 49(2): 177–82.

22. Perrin M, Vandeleur CL, Castelao E, *et al.* Determinants of thedevelopment of post-traumatic stress disorder, in the general population. *Soc Psychiatry Psychiatr Epidemiol*2014; 49(3): 447-457.

23. Holbrook TL, Hoyt DB, Stein MB,*et al.* Gender differences in long-term post-traumatic stress disorder outcomes after major trauma: women are at higher risk of adverse outcomes than men. *J Trauma* 2002; 53(5):882-888.

24. Yuemei X, Fenglin C. Post-traumatic stress disorder symptoms and its relation with Quality of life in Ambulance Nurses of Linyi. *Shandong University Master's Thesis*2010.

25. Lulu C, Min L. Related investigation on Post Traumatic Stress Disorder, Mental Health,

For peer review only - http://bmjopen.bmj.com/site/about/guidelines.xhtml

Resilience, Personality and Coping Style of Gynecologic Cancer Patients. *Third Military Medical University*2012.

26. Chengqi L, Guorui L. A study on the relationship between College Students' attribution style and their mental health. *East China Normal University* 2004.

27. ILO/ICN/WHO/PSI. Framework Guidelines for Addressing Workplace Violence in the Health Sector. Geneva: ILO/ICN/WHO/PSI Joint Programme on Workplace Violence in the Health Sector; 2002.

28. Zampieron A, Galeazzo M, Turra S, *et al.* Perceived aggression towards nurses: study in two Italian health institutions. *J Clin Nurs* 2010;19:2329–41.

29. Weathers FW, Litz BT, Herman DS, *et al.* The PTSD Checklist (PCL):Reliability, validity,and diagnostic utility. Annual convention of the International Society for Traumatic Stress Studies. *San Antonio* TX; 1993.

30. Zhang Z, Shi Z, Wang L, *et al.*Post-traumatic stress disorder, anxiety and depression among the elderly: a survey of the hard-hit areas a year after the Wenchuan earthquake. *Stress Health* 2012; 28(1):61-68.

31. Wu Z, Xu J, Sui Y. Posttraumatic stress disorder and posttraumatic growth coexistence and the risk factors in Wenchuan earthquake survivors. *Psychiatry Res* 2016; 237:49-54.

32. Li L, Yang Y L, Wang Z Y, et al. Prevalence and Positive Correlates of Posttraumatic Stress Disorder Symptoms among Chinese Patients with Hematological Malignancies: A Cross-Sectional Study. *Plos One* 2015;10(12):e0145103.

33. Cui Y, Tian SS, Qiao N, *et al.* Associations of Individual-Related and Job-Related Risk Factors with Nonfatal Occupational Injury in the Coal Workers of Shanxi Province: A Cross-Sectional

For peer review only - http://bmjopen.bmj.com/site/about/guidelines.xhtml

Study. *PLoS One* 2015;10(7):e0134367.

34. Qian M, Wu G, Zhu R, *et al.* Development of the revised Eysenck Personality Questionnaire Short Scale for Chinese (EPQ-RSC). *Acta Psychologica Sinica* 2000;32(3):317-323.

35. Zhang L, Zhang J X, Wen-Juan X U, *et al.* An evaluation of validity and reliability on the trait coping style questionnaire(TCSQ) using for Chinese soldier. *Chinese Journal of Disease Control & Prevention*2010.

36. Zhao Q, Wenjuan X U, Jing L U, *et al.* Study on the relationship between the trait coping style and the quality of life of soldiers in Xinjiang area.*Journal of Shanxi Medical University*2012.

37. Feng S, Tan H, Benjamin A, *et al.* Social support and posttraumatic stress disorder among flood victims in Hunan, China. *Ann Epidemio*l. 2007;17(10):827-833.

38. Li H, Yan C, Zhu S, *et al.* Correlation among coping style, social support, and negative emotion in infertile women. Zhong nan da xue xue bao. Yi xue ban = Journal of Central South University. *Medical sciences* 2011;36(2):138-142.

39. Chang-Fei L U, Jia C X, Zhang J Y, *et al.* Reliability and validity of Social Support Rating Scale in rural suicides. *Chinese Mental Health Journal* 2011;25(3):218-222.

40. Zafar W, Khan UR, Siddiqui SA,*et al.*Workplace Violence and Self-reported Psychological Health: Coping with Post-traumatic Stress, Mental Distress, and Burnout among Physicians Working in the Emergency Departments Compared to Other Specialties in Pakistan.*J Emerg Med* 2015;50(1):167-177.

41. Johansen VA, Eilertsen DE, Nordanger D, Weisaeth L. Prevalence, comorbidity and stability of post-traumatic stress disorder and anxiety and depression symptoms after exposure to physical assault: an 8-year prospective longitudinal study. *Nord J Psychiatry*2013; 67(1):69-80.

For peer review only - http://bmjopen.bmj.com/site/about/guidelines.xhtml

42. Breslau N. The epidemiology of post-traumatic stress disorder; what is the extent of the problem? *Journal of Clinical Psychiatric* 2001;62:16-22.

43. Alden LE, Regambal MJ, Laposa JM. The effects of direct versus witnessed threat on emergency department healthcare workers: Implications for PTSD criterion A. *J Anxiety Disord*2008;22(8): 1337-1346.

44. O'Leary M M, Taylor J, Eckel L. Psychopathic personality traits and cortisol response to stress: the role of sex, type of stressor, and menstrual phase.*Hormones & Behavior* 2017;58(2):250-256.

45. Cheung T, Yip PSF. Depression, anxiety and symptoms of stress among Hong Kong nurses: a cross-sectional study. *Inter J Env Res Pub Heal* 2015;12(9):11072-11100.

For peer review only

!

1. the National Natural Science Foundation of China, grant number71473063

国家自然科学基金委员会
项 目 批 准 通 知

国科金计项〔2014〕44号

关于批准资助2014年度第二批项目的通知

哈尔滨医科大学（单号：2014-44-0330）：

根据《国家自然科学基金条例》有关规定和专家评审意见，国家自然科学基金委员会（以下简称自然科学基金委）决定批准资助你单位2014年度（第2批）国家自然科学基金项目 125 项，金额 5902.0 万元。其中，面上项目 51 项，重点项目 2 项，青年科学基金项目 72 项，上述资助项目清单详见附件。

自评审结果通告发布之日起25日内，项目负责人须按要求填写与提交《国家自然科学基金资助项目计划书》（以下简称计划书）电子版。2014年9月11日16点前，依托单位将审核后的计划书电子版通过科学基金网络信息系统（https://isis.nsfc.gov.cn）提交至自然科学基金委。自然科学基金委同期对计划书电子版进行审核。审核通过的，项目负责人可打印计划书纸质版（建议双面打印）；审核未通过的，退回至项目负责人修改，依托单位须在2014年9月18日16点前，将修改后的计划书电子版及时审核并再次提交至自然科学基金委。2014年9月26日16点前，依托单位须将自然科学基金委审核通过后的计划书纸质版（一式两份，应保证与电子版一致）加盖单位公章，报送至自然科学基金委项目材料接收工作组。采用邮寄方式的，请在截止日前（以发信

邮戳日期为准）以快递方式邮寄，并在信封左下角注明“计划书”。请勿使用包裹，以免延误报送。报送计划书材料时，还应包括本单位报送计划书的公函和计划书清单。材料不完整不予接收。

如在规定期限内未提交电子与纸质计划书且未说明理由的，视为自动放弃接受资助。

邮寄地址：北京市海淀区双清路83号项目材料接收工作组
邮编：100085
联系电话：010-62328591

附件：2014年度国家自然科学基金资助项目清单

国家自然科学基金委员会

国家自然科学基金委员会
2014年8月25日

2014年国家自然科学基金资助项目清单（哈尔滨医科大学）

单号：2014-44-0330

资助金额单位：万元

序号	项目批准号	负责人	申请代码	项目名称	资助金额	起止日期	资助类别/亚类说明/附注说明
12	61403112	刘洪波	F030406	基于高通量组学数据的干细胞多能性相关DNA甲基化调控元件识别及其功能分析	25	2015.01.01-2017.12.31	青年科学基金项目
13	61473106	李霞	F030407	癌症非编码miRNA-lncRNA协同调控网络重构与功能特性研究	82	2015.01.01-2018.12.31	面上项目/常规面上项目
14	71403071	孙涛	G0308	基于生态理论视角下区域医疗联合体生态系统健康评价建模与实证研究	23	2015.01.01-2017.12.31	青年科学基金项目
15	71403072	王月枫	G0308	社区高血压患者急症风险预测模型构建及预见性干预与救护管理体系的研究	20	2015.01.01-2017.12.31	青年科学基金项目
16	71403073	李叶	G0308	基于CHE方法学比较分析的医保制度经济保护能力影响因素研究	23	2015.01.01-2017.12.31	青年科学基金项目
17	71403074	尹慧	G0308	基于结构方程的社区心血管疾病的社会决定因素模型构建及分层干预效果评价	20	2015.01.01-2017.12.31	青年科学基金项目
18	71473063	樊立华	G0308	医护人员遭受医院场所暴力PTSD及对职场行为影响机制研究	63	2015.01.01-2018.12.31	面上项目/常规面上项目
19	71473064	焦明丽	G0308	基于DEMATEL 法与“破窗理论”的医院暴力影响因素及对策研究	60	2015.01.01-2018.12.31	面上项目/常规面上项目
20	71473065	宁宁	G0308	面向公共卫生灾害的城市社区抗逆力理论、方法及策略研究	63	2015.01.01-2018.12.31	面上项目/常规面上项目
21	81400097	高春艳	H0811	血管内皮细胞清除活化和凋亡的血小板调控血栓形成的作用与机制	23	2015.01.01-2017.12.31	青年科学基金项目
22	81400115	付玥玥	H0812	APL分化综合征miRNA标记筛选及功能分析	23	2015.01.01-2017.12.31	青年科学基金项目

第2页 / 共12页

2. Ethical Approval:

哈尔滨医科大学公共卫生学院医学伦理审查申请表

项目名称：医护人员遭受医院暴力 PTSD 及对职场行为影响机制研究	
项目负责人：樊立华	职称：教授
电话：13136661393	电子信箱：lihuafan@126.com
所在单位:哈尔滨医科大学公共卫生学院	
单位伦理委员会意见：<br><br>按照国家、省有关部门医学伦理审查的规定和程序，哈尔滨医科大学公共卫生学院医学伦理委员会对本申请的项目审查结论为：研究项目所涉及的医学伦理内容和实施的解决方案符合要求。	
伦理委员会成员签字：	
伦理委员会主任签字：　　　单位伦理委员会公章：<br>2014年 3月 1日	

哈尔滨医科大学医学伦理委员会

1

Checklist of ***cross-sectional studies***

	Page number, line number
**Title**	1, 4-6
**Abstract**	2, 4-57; 3, 32
**Strengths and limitations of this study**	3, 37-57
**Introduction**	
Background	4, 4-57; 5, 4-34
Objectives	5, 16-24
**Methods**	
Study design	5, 39-57; 6, 4-42
Setting	5, 52-57; 6, 4-19
Participants	6, 31-42
Questionnaire	6, 47-57; 7; 8; 9, 4-32
Data measurement	9, 36-57; 10, 4-14
Study size	6, 24-29
Quantitative variables	7, 47-57
Ethical considerations	10, 19-29
**Results**	
Participants	10, 36-44
Descriptive data	10, 36-58; 11, 3-43
Outcome data	12
Main results	11; 12;13;14
Other analyses	16, 37-53; 17,3-32
**Discussion**	
Key results	14,38-57; 15, 4-56
Interpretation	16, 3-32; 17, 9-57
Discuss the external validity of the study results	17, 19-31
**Conclusions**	17, 36-52
**Other information**	
Acknowledgements	18, 4-6
Contributors	18, 9-14
Funding	18, 16-19
Competing interests	18, 21
**References**	18, 29-57; 20; 21; 22; 23;24

For peer review only

BMJ Open

Prevalence and correlates of symptoms of post-traumatic stress disorder among Chinese healthcare workers exposed to physical violence: a cross-sectional study

Journal:	*BMJ Open*
Manuscript ID	bmjopen-2017-016810.R1
Article Type:	Research
Date Submitted by the Author:	13-May-2017
Complete List of Authors:	Shi, Lei<br>Wang, Lingling ; Chinese Hospital Association, Department of Autonomous Protection<br>Jia, Xiaoli<br>Li, Zhe<br>Mu, Huitong<br>Liu, Xin<br>Peng, Boshi<br>Li, Anqi<br>Fan, Lihua; Harbin Medical University, Department of Health Management , School of Public Health
**Primary Subject Heading**:	Health policy
Secondary Subject Heading:	Health policy, Health services research, Mental health
Keywords:	Posttraumatic stress disorder symptoms (PTSD), physical violence, social support, coping styles, MENTAL HEALTH, personality

SCHOLARONE™
Manuscripts

Prevalence and correlates of symptoms of post-traumatic stress disorder among Chinese healthcare workers exposed to physical violence: a cross-sectional study

Lei Shi,[1] Lingling Wang,[2] Xiaoli Jia,[2] Zhe Li,[1] Huitong Mu,[1] Xin Liu,[1] Boshi Peng,[3] Anqi Li,[1] Lihua Fan[1]

Correspondence to: Lihua Fan, School of Public Health, Harbin Medical University, 157 Baojian Road, Nangang District, Harbin 150081, China; E-mail: lihuafan@126.com

[1]Department of Health Management, School of Public Health, Harbin Medical University, Harbin, Heilongjiang, China

[2]Department of Autonomous Protection, Chinese Hospital Association, Beijing, China

[3] Department of Scientific Research, The Second Affiliated Hospital of Harbin Medical University, Harbin, Heilongjiang, China

For peer review only

For peer review only - http://bmjopen.bmj.com/site/about/guidelines.xhtml

Abstract

Objectives

Post-traumatic stress disorder (PTSD) is a common psychological maladjustment to undergoing a traumatic event. Our aim was to measure the prevalence of PTSD among Chinese healthcare workers exposed to physical violence, and explore the associations of their demographic characteristics, social support, personality traits, and coping styles with their PTSD symptoms.

Methods

A cross-sectional study was conducted using the: Workplace Violence Scale, Posttraumatic Stress Disorder Checklist-Civilian Version (PCL-C), Social Support Rating Scale (SSRS), Revised Eysenck Personality Questionnaire-Short Scale and Trait Coping Style Questionnaire. We used convenience sampling method to collect data from March 2015 to September 2016. Healthcare workers (N = 2,706) from 39 public hospitals located in Heilongjiang, Hebei, and Beijing Provinces of China completed the questionnaires (effective response rate = 84.25%).

Results

Overall, the prevalence of physical violence in the previous 12 months was 13.60% (N = 2,706).The prevalence of PTSD among the healthcare workers who experienced physical violence was 28.0% (n = 368). Most of the victims of physical violence (50.80%) did not exhibit PTSD symptoms based on their PCL-C scores, and 47.0% did not manifest the diagnostic criteria for PTSD after experiencing physical violence. The level of PTSD symptoms was negatively correlated with their scores on the SSRS ($r = -0.188$, $P < 0.001$). The hierarchical regression analysis (Block 3) revealed that in women, positive coping was significantly associated with PTSD symptoms ($\beta = -0.376$, $P = 0.001$). However, the effect of positive coping was not significant in men.

For peer review only - http://bmjopen.bmj.com/site/about/guidelines.xhtml

Conclusions

The prevalence of PTSD among the victims was similar to that found in Atlanta. The positive effects of social support on PTSD symptoms suggest that it has practical implications for interventions to promote psychological health. The healthcare workers' coping styles influenced the development of PTSD symptoms. Therefore, adopting effective coping styles and receiving social support have potential roles in the recovery from trauma after experiencing physical violence.

Strengths and limitations of this study

- In China, few studies have been conducted on PTSD symptoms following healthcare workers' exposure to physical violence.
- We assessed the prevalence of PTSD and explored the correlates of PTSD symptoms among Chinese healthcare workers exposed to physical violence.
- Our study was conducted at 39 public hospitals in three provinces using convenience sampling. Therefore, the representativeness of the sample is limited.
- The retrospective approach to collecting data using self-reports of PTSD symptoms might have led to recall and report bias.

For peer review only - http://bmjopen.bmj.com/site/about/guidelines.xhtml

BACKGROUND

Post-traumatic stress disorder(PTSD) is a psychological state of imbalance, characterized by a series of chronic emotional reactions to a traumatic event, including re-experiencing, avoidance, and heightened arousal, as outlined in the Diagnostic and Statistical Manual of Psychiatric Disorders-4th edition (DSM-IV).[1-3] However, the criteria for PTSD in the manual's fifth edition (DSM-5) include not three but four symptom clusters: including re-experiencing, avoidance, negative alterations in mood and cognition, and hyperarousal.[4] It is worth noting that PTSD has shifted from its classification as an anxiety disorder in the DSM-IV to a new category of Trauma and Stress-related Disorders in the DSM-5.[4] Although a substantial number of studies indicate that almost all people exhibit intrusive and repetitious symptoms after exposure to excessive stress,[5]only a small percentage develop avoidance and hyper-arousal symptoms. Most individuals showing PTSD symptoms after exposure to a traumatic event recover within weeks or months. However, 10%–25% might develop chronic PTSD that lasts for several months or years, or even a lifetime.[6]

PTSD originated from reports of the war-related trauma, and was applied gradually to a variety of man-made and natural disasters.[7] Scholars have reported that the incidence of PTSD among male and female Vietnam veterans in the USA is 15.2% and 8.5%, respectively.[8] Moreover, most of the Chinese studies on PTSD have focused on wars, traffic accidents, and natural disasters.[9-10] Differences in the incidence rates of PTSD for different types of trauma have been reported in China. For instance, the prevalence of PTSD has been reported to be 8.65% among soldiers assigned to military vehicles at high altitudes, 33.89% among flood-disaster survivors, 18.8% among earthquake survivors, 41% among traffic-accident survivors, and 78.6% among survivors of a serious explosions.[11]

PTSD symptoms and the full range of criteria comprising a PTSD diagnosis have been observed

For peer review only - http://bmjopen.bmj.com/site/about/guidelines.xhtml

in rescue and ambulance personnel.[12-13] Healthcare workers typically are exposed to two types of trauma in the hospital setting: direct (personal involvement in traumatic events through confrontations resulting in their own traumatic experiences e.g., workplace violence) and indirect (non-personal involvement in traumatic events through others' confrontations resulting in other people's traumatic experiences e.g., witnessing other people's direct experiences of workplace violence, caring for dying patients, and threats of severe injury or exposure to trauma).[4 14-16] In the present study, a traumatic event refers to a healthcare worker's exposure to physical violence in the workplace. Workplace violence is divided into physical and psychological violence.[17] Physical violence causes more serious physical and psychological damage (e.g., PTSD, anxiety, fear, and depression) than other forms of violence.[18-20] Physical violence refers to the use of physical force against an individual or a group, and can lead to physical, psychological, or sexual harm; it includes hitting, shooting, kicking, slapping, pushing, biting, pinching, wounding using sharp objects, and sexual assault and rape.[17] Approximately 50% of healthcare workers have experienced at least one violent incident during their working lives.[21] During the past 12 months, the incidence rate of physical violence for nurses in different countries has ranged from 9.1% to 56.0%.[22-25]The results of a systematic review of studies conducted in Iran indicated that the most common types of physical violence experienced by 43% of participants were pushing or pinching.[26] In China, physician-patient conflicts present a growing trend, with an increase in the number of healthcare workers killed by patients or their relatives to 24, and an increase in injures from 2003 to 2013.[27] Several studies have estimated the prevalence of PTSD among emergency department staff to range from 10% to 25%.[28-30] Noelle Robertson and Andrew Perry conducted a systematic review of PTSD research investigations; the results showed that the prevalence of PTSD ranged from 8% to 29% among different hospital-based departments.[31] There are also reports of the occurrence of PTSD

For peer review only - http://bmjopen.bmj.com/site/about/guidelines.xhtml

among Chinese nurses working in emergency departments, intensive care units, and operating rooms. However, the number of research studies on PTSD among healthcare workers has been relatively few in China.

Demographic variables (e.g., age, gender, and educational level)and psychological and social variables (e.g., personality, coping style, and social support) have been found to be significantly associated with cancer-related PTSD symptoms.[32-33] Previous studies have found that the risk of PTSD was most strongly associated with neuroticism and problem-focused coping strategies in the general population.[34-35] Neuroticism was the most critical personality dimension in predicting PTSD, and avoidant coping and social support mediated the relationship between neuroticism and PTSD in a high proportion of adult burn survivors.[36] Social support has been reported to play a significant role in helping nurses cope with work-related stress.[37] A meta-analysis indicated that work-related critical incidents were positively related to PTSD symptoms.[38]

In this study, we aimed to assess the prevalence of PTSD, and to explore the associations of demographic characteristics, social support, personality characteristics, and coping styles with PTSD symptoms among Chinese healthcare workers exposed to physical violence.

METHODS

Participants and Procedures

A cross-sectional study was conducted from March 2015 through September 2016 with a sample of healthcare workers employed by 39 public hospitals located in Heilongjiang, Hebei, and Beijing Provinces of China. The 39 public hospitals that served as the research settings were chosen using convenience sampling method (convenience sampling method is a non-probability method, and the

For peer review only - http://bmjopen.bmj.com/site/about/guidelines.xhtml

findings should not be generalized). All investigators were trained using a uniform survey manual before they began to collect data. Qualified investigators were appointed to collect data. We obtained permission from the managers and the medical dispute resolution and human resources departments of the hospitals. The investigators conducted surveys by using an anonymous, self-administered questionnaire. We purposely selected 3 public hospitals in Harbin (the First Affiliated Hospital of Harbin Medical University, the Second Affiliated Hospital of Harbin Medical University, and the Fourth Affiliated Hospital of Harbin Medical University) as the sites for our pilot study site before the formal investigation. A total of 150 questionnaires were distributed and returned (these data were excluded from the main study). A total of 3,212 healthcare workers (i.e., physicians, nurses and medical technicians) were investigated using convenience sampling in the formal investigation. The researchers and hospital coordinators distributed and collected the questionnaires that were completed by the healthcare workers immediately. A total of 2,706 valid questionnaires were returned, and the effective response rate was 84.25%.This study's focus was only on PTSD symptoms among healthcare workers exposed to physical violence; thus, only 368 responses were considered valid data and were analyzed in the present study.

The inclusion criteria for participation in this study were as follows: (1) at least one year of work experience; (2) voluntary participation; (3) participation would not affect the participation's work; and (4) experience of physical violence in the previous 12 months. Individuals were excluded if they (1) had received any psychological treatment after experiencing physical violence; (2) experienced other traumatic events, including workplace psychological violence or serious life events (e.g., domestic violence or attacks by criminals), serious accidents (e.g., fires, explosions, or traffic accidents), natural disasters (e.g., typhoons, earthquakes, or floods), or (3) were indirectly exposed to trauma,[39-40] (e.g.,

For peer review only - http://bmjopen.bmj.com/site/about/guidelines.xhtml

witnessing other people experience traumatic events).

QUESTIONNAIRE

Demographic characteristics

Demographic data on the healthcare workers were collected, including gender, age, marital status, educational status, professional title, department, occupation, and work experience. Age was categorized as ≤30, 31−50, and ≥51 years old. Marital status was categorized as married and single/ divorced/widowed. Educational status was classified as junior college or below, undergraduate, and graduate. Occupation was divided into three groups: physician, nurse, and medical technician. Professional title was categorized as primary, intermediate, and senior. Department was classified as emergency department, internal medicine, surgery, obstetrics and gynecology, pediatrics, and other. Work experience was divided into four categories: ≤ 4, 5–10, 11–20, and ≥ 21 years.

Workplace Violence Scale

The Workplace Violence (WPV) Scale developed by the International Labour Office, International Council of Nurses, World Health Organization, and Public Services International Joint Programme on Workplace Violence in the Health Sector in 2003and the revised Survey of Violence Experienced by Staff (SOVES-G-R) was used to measure workplace violence.[41-42] We obtained permission to use these scales. The scale used in this study consists of 2 dimensions (physical violence and psychological violence) and has 9 items that were adopted from these scales. Each item is scored on a 4-point scale reflecting respondents' frequency of exposure to violence in the past 12 months (0 = 0 times, 1 = 1 time, 2 = 2–3 times, and 3 = ≥4 times). The total possible score ranges from 0 to 27, with a higher total score indicating a higher frequency of exposure to WPV. The physical violence subscale consists of 6 items, thus, the total possible score ranges from 1 to 18. In the present study, Cronbach's α for the WPV Scale

For peer review only - http://bmjopen.bmj.com/site/about/guidelines.xhtml

was 0.86.

PTSD

The PTSD Checklist-Civilian Version (PCL-C),which has been used to measure PTSD symptoms among healthcare workers was used in the present study.[43-44] It consists of 17 self-report items, which comprise three dimensions, namely, re-experiencing (items 1–5), avoidance/numbing (items 6–12) and hyper-arousal (items 13–17). The three dimensions correspond to the DSM-IV symptoms criteria for PTSD.[2] The response options for each item on the PCL-C are rated from 1 (not at all) to 5 (extremely), based on the extent to which the respondent has been troubled by specific symptoms in the past month. The total possible score is calculated by adding the scores for all items, and it ranges from 17 to 85 points, with a higher score indicating a higher risk for PTSD symptoms. A total score≥ 50 is indicative of the full PTSD diagnosis (sensitivity = 0.82; specificity = 0.83; kappa = 0.64).[45] In this study, the traumatic event in the original PCL-C was replaced by physical violence. The reliability and validity of this instrument have been shown to be high in a wide range of Chinese samples.[46] The present study revealed that Cronbach's α for the PCL-C was 0.934, and for the three subscales it was 0.872 (re-experiencing), 0.921 (avoidance), and 0.926 (hyper-arousal).

Revised Eysenck Personality Questionnaire-Short Scale

Personality traits were measured using the **Revised** Eysenck Personality Questionnaire-**Short Scale** for Chinese (EPQ-RSC).[47-48] The EPQ-RSC consists of 48 items, categorized into 4 subscales reflecting personality traits: Extraversion, Neuroticism, Psychoticism, and Lie. Each item is scored on a dichotomous scale (1=Yes, 0=No) to measure personality characteristics. The scores of the positively and negatively worded items are summed in accordance with each personality trait. Early studies found the EPQ-RSC to have high reliability and validity as a measure of personality traits inChina.[48 -49] The

For peer review only - http://bmjopen.bmj.com/site/about/guidelines.xhtml

total score for the Extraversion subscale indicates introversion when it is less than 43.3, intermediate when it is from 43.3 to 56.7 and extraversion when it is greater than 56.7.[49] For the Psychoticism subscale, tough-minded is defined as a total score greater than 56.7; intermediate is defined as a total score is between 43.3 and 56.7, and mild is defined as a total score less than 43.3.[49] For the Neuroticism subscale, a total score of less than 43.3 defines emotional stability, whereas a total score from 43.3 to 56.7 defines intermediate, and a total score greater than 56.7 defines emotional instability.[49] For the Lie subscale, a total score of 60 or greater indicates that information provided by the respondent might beunreliable.[49] In this study, Cronbach'sαfor the EPQ-RSC was 0.903. The internal consistency coefficients were 0.854, 0.756, 0.791, and 0.762, for the Extraversion, Neuroticism, Psychoticism, and Lie subscales, respectively.

Trait Coping Style Questionnaire

The Trait Coping Style Questionnaire (TCSQ) was used in this study to assess participants' style of coping with life events.[37 50] The TCSQ consists of 20 items, including 10 items measuring positive coping (items 1, 3, 5, 8, 9, 11, 14, 15, 18, 20) and 10 items measuring negative coping (items 2, 4, 6, 7, 10, 12, 13, 16, 17, 19). Positive coping refers to individuals who, when faced with a problem, tend to deal with it in a positive way, and are able to quickly forget unpleasant aspects. Negative coping refers to the tendency to use negative coping methods to deal with problems and vent frustrations to other people, which makes it is easier to ignore unpleasant thoughts. For example, when conflicts with others, arise, individuals who use negative coping will ignore the opposing side for a long time.[51] Each item is rated on a 5-point Likert scale. The total possible score for positive and negative coping is calculated by adding the scores for all the items. Previous studies have found the TCSQ to have high reliability and validity as a measure of coping style inChina.[50-51] In this study, Cronbach's α for the total scale was

For peer review only - http://bmjopen.bmj.com/site/about/guidelines.xhtml

0.845, and the internal consistency coefficients of the subscales were α=0.823 (positive coping), and α=0.863 (negative coping).

Social Support Rating Scale

Social support was evaluated using the Chinese version of the Social Support Rating Scale (SSRS),[52-54] which is a brief measure of the overall situation of respondents' social support. This 10-item scale is divided into 3 dimensions: subjective support (items 1, 3, 4, 5), objective support (items 2, 6, 7) and utilization of support (items 8, 9, 10). Subjective support refers to an individual's emotional experience of being respected, supported, and understood by their social group, and it is closely related to the individual's subjective feelings. Objective support refers to visible support, including material and direct assistance, social networks, group relationships, and the individual's degree of participation in societal activities with family, friends, and colleagues (e.g., marriage). A low level of social support is defined as a total score between 12 and 44, an intermediate level as a total score is between 45 and 54, and high level as a total score greater than 55.[54] The present study revealed that Cronbach's α for the SSRS was 0.865, and for the three subscales it was 0.884 (subjective support), 0.911(objective support), and 0.875 (the availability of support).

DATA ANALYSIS

EpiData version 3.1 was used to establish the study's database. We eliminated the questions with missing data or quality issues. To ensure accuracy, two trained personnel entered the data after all the surveys were completed. IBM SPSS Version 19.0 and Excel were used for the data analysis. The normal distributions of the continuous variables were verified using P-P plots and K-S tests. Descriptive statistics, including numbers (n), percentages (%), means, and standard deviations (SD)

For peer review only - http://bmjopen.bmj.com/site/about/guidelines.xhtml

were calculated for the demographic variables. We used one-way analysis of variance (ANOVA) or independent sample t-tests to compare group differences on the measures of the continuous variables. The chi-square (χ^2) test was used to compare differences in the categorical variables. Pearson's correlations were used to examine correlations among the continuous variables. Hierarchical regression analysis was used to examine the associations of the demographic characteristics and the scores on the SSRS, EPQ-RSC, and TCSQ with PTSD symptoms. Statistics, including F values, R^2, R^2-changes (ΔR^2), standardized regression coefficients (β), and P-values for each step in the regression model were reported. All the study variables were tested for multicolinearity. A P-value < 0.05 was considered to be statistically significance.

STROBE STATAMENT

We declared that the STROBE (Strengthening the Reporting of Observational Studies in Epidemiology) guidelines are followed in this study.

ETHICAL CONSIDERATIONS

Ethical approval to conduct this study was granted by the Research Ethics Committee of Harbin Medical University, and informed consent was obtained from each hospital and healthcare worker involved in the investigation. All of the participants gave their informed consent before the survey; they were assured that their personal information would be kept confidential.

RESULTS

Demographic Characteristics of the Respondents

For peer review only - http://bmjopen.bmj.com/site/about/guidelines.xhtml

The demographic characteristics of the respondents are shown in Table 1.

Table 1. Demographic characteristics of the respondents (N=2706).

Demographic variables		n	%
Gender	Male	623	23.0
	Female	2083	77.0
Age group	≤ 30	1258	46.5
	31-50	1341	49.5
	≥ 51	107	4.0
Educational level	Junior college or below	856	31.6
	Undergraduate	1341	49.6
	Graduate	509	18.8
Marital status	Married	1859	68.7
	Single/divorced/widowed	847	31.3
Occupation	Physician	1058	39.1
	Nurse	1520	56.2
	Medical Technician	128	4.7
Technical title	Primary	1147	42.4
	Intermediate	1026	37.9
	Senior	533	19.7
Department	Emergency Department	323	11.9
	Internal Medicine	813	30.0
	Surgery	752	27.8
	Obstetrics and Gynecology	276	10.2
	Pediatrics	218	8.1
	Other	324	12.0
Years of experience	≤ 4	1014	37.5
	5-10	820	30.3
	11-20	503	18.6
	≥ 21	369	13.6

Characteristics of the Victims in Relation to PTSD Symptoms

Of the 368 victims of physical violence, 59.8% were women, 51.3% completed an undergraduate education, and 73.9% were married. The characteristics of the victims in relation to PTSD symptoms are presented in Table 2.

Table 2. Characteristics of victims in relation to PTSD symptoms. (N=368).

Variables		n	%	PTSD symptoms		F/t	*P*
				Mean	SD		
Gender	Male	148	40.2	44.03	16.19	3.537	0.000
	Female	220	59.8	38.30	13.71		

For peer review only - http://bmjopen.bmj.com/site/about/guidelines.xhtml

Age group	≤ 30	133	36.1	38.09	13.36	2.946	0.054
	31-50	216	58.7	42.01	15.56		
	≥ 51	19	5.2	42.10	17.80		
Educational level	Junior college or below	118	32.1	38.14	13.54	2.592	0.076
	Undergraduate	189	51.3	42.13	15.46		
	Graduate	61	16.6	40.64	15.85		
Marital status	Married	272	73.9	41.51	15.75	2.195	0.029
	Single/divorced/widowed	96	26.1	38.03	12.38		
Occupation	Physician	175	47.6	42.97	15.37	4.379	0.013
	Nurse	180	48.9	38.29	13.82		
	Medical Technician	13	3.5	40.69	21.24		
Technical title	Primary	145	39.4	39.56	13.04	0.576	0.562
	Intermediate	126	34.2	41.32	16.21		
	Senior	97	26.4	41.23	16.16		
Department	Emergency Department	68	18.5	41.46	16.08	0.722	0.607
	Internal Medicine	76	20.7	38.45	15.07		
	Surgery	123	33.4	41.53	13.91		
	Obstetrics and Gynecology	19	5.2	41.63	16.52		
	Pediatrics	27	7.3	37.63	10.37		
	Other	55	14.9	41.55	17.28		
Years of experience	≤ 4	101	27.4	37.19	13.25	2.158	0.063
	5-10	120	32.6	42.13	14.52		
	11-20	87	23.7	41.90	16.80		
	≥ 21	60	16.3	41.42	15.46		
Social support	Low	224	60.9	42.41	15.06	5.904	0.003
	Medium	130	35.3	38.52	14.40		
	High	14	3.8	31.00	14.53		
Subjective support	Low	22	6.0	41.91	18.63	0.859	0.425
	Medium	38	10.3	43.37	12.93		
	High	308	83.7	40.17	14.97		
Objective support	Low	206	56.0	42.19	15.47	3.369	0.035
	Medium	155	42.1	38.87	14.32		
	High	7	1.9	32.00	9.24		
Utilization of support	Low	39	10.6	48.18	16.98	6.979	0.001
	Medium	259	70.4	40.37	14.56		
	High	70	19.0	37.24	14.23		
Extraversion	Introversion	102	27.7	39.45	13.35	1.278	0.280
	Middle	164	44.6	41.99	16.04		
	Extraversion	102	27.7	39.51	14.80		
Psychoticism	Mild	68	18.5	42.22	16.87	0.998	0.370
	Middle	213	57.9	40.79	15.24		
	Tough-minded	87	23.6	38.86	12.69		
Neuroticism	Emotional instability	100	27.2	40.33	13.80	0.530	0.589
	Middle	153	41.6	41.50	16.72		

For peer review only

Emotional stability	115	31.2	39.63	13.60

Note. PTSD = post-traumatic stress disorder; SD = standard deviations.

Prevalence of Physical Violence in the Previous 12 Months

During the past 12 months, the prevalence of physical violence and psychological violence toward healthcare workers were 13.60% (368/2706) and 59.64% (1614/2706), respectively. The respondents reported that the patients' relatives were the main perpetrators (67.4%, n = 248), followed by the patients (23.6%, n = 87).

Prevalence of PTSD

The PTSD symptoms based on the victims' PCL-C scores are summarized in Table 3. According to their scores on the PCL-C, 103 victims (28.0%) met the full criteria for a PTSD diagnosis and 21.2% of victims were at risk for developing PTSD.

According to the DSM IV-TR criteria for PTSD,[2] 47.0% of the victims did not appear to manifest the diagnostic criteria. Re-experiencing was the most frequently observed criterion for PTSD observed among the victims (45.1%), followed by hyper-arousal (37.8%).

Table 3. Sample description and prevalence of PTSD.

PTSD symptoms	Physical violence	
	n	%
**PTSD symptoms based on PCL-C scores**		
No obvious PTSD symptoms (17-37)	187	50.8
Criteria met for potential risk of PTSD (38-49)	78	21.2
Criteria met for the full PTSD diagnosis (50-85)	103	28.0
**PTSD symptoms based on PTSD criterion***		
No criterion manifestation	173	47.0
Re-experiencing (Criterion B)	166	45.1
Avoidance (Criterion C)	129	35.1
Hyper-arousal (Criterion D)	139	37.8

Note. PTSD = post-traumatic stress disorder; PCL-C = PTSD Checklist-Civilian Version.
*Participants may have more than one criteria.

Correlations of the EPQ-RSC, TCSQ and SSRS Scores with PTSD Symptoms

Table 4 shows the correlations among the victims' PTSD symptoms and scores on the EPQ-RSC,

For peer review only - http://bmjopen.bmj.com/site/about/guidelines.xhtml

Table 4. Pearson correlations among PTSD symptoms, EPQ-RSC, TCSQ, SSRS and Physical Violence.

Variables	Mean	SD	1	2	3	4	5	6	7	8	9	10	11	12	13	14
1.PTSD symptoms	40.60	15.01	-													
2.Re-experiencing	12.43	4.92	0.89**	-												
3.Avoidance	15.70	5.99	0.94**	0.77**	-											
4.Hyper-arousal	12.46	5.48	0.91**	0.70**	0.79**	-										
5.Physical violence	3.08	2.99	0.26**	0.22**	0.31**	0.18**	-									
6.SSRS	41.73	8.44	-0.19**	-0.12**	-0.22**	-0.17**	-0.12**	-								
7.Subjective support	25.23	5.14	-0.09	-0.02	-0.13*	-0.09	0.01	0.89**	-							
8.Objective support	8.63	3.30	-0.21**	-0.17**	-0.23**	-0.17**	-0.20**	0.77**	0.46**	-						
9.Utilization of support	7.87	2.01	-0.21**	-0.15**	-0.22**	-0.20**	-0.21**	0.66**	0.43**	0.40**	-					
10.Positive coping of TCSQ	30.05	7.22	-0.16**	-0.15**	-0.12*	-0.18**	-0.13*	0.10	0.04	0.10*	0.15**	-				
11.Negative coping of TCSQ	26.92	7.33	0.19**	0.10	0.19**	0.22**	0.04	-0.31**	-0.26**	-0.29**	-0.17**	0.12*	-			
12.Extraversion	49.81	10.33	-0.01	-0.03	0.01	-0.01	0.06	-0.05	-0.02	-0.08	-0.02	-0.04	0.02	-		
13.Psychoticism	50.10	9.81	0.06	0.06	0.06	0.04	0.00	0.02	0.02	0.06	0.02	0.02	0.04	-0.02	-	
14.Neuroticism	50.07	10.34	0.03	0.04	0.01	0.05	0.03	0.03	0.01	0.05	0.04	0.01	0.04	-0.09	0.20**	-

Note. SD = standard deviations; PTSD = post-traumatic stress disorder; EPQ-RSC = Eysenck Personality Questionnaire-Revised Short Scale for Chinese; SSRS = Social Support Rating Scale; TCSQ = Trait Coping Style Questionnaire.

$^{*}P<0.05$, $^{**}P<0.01$

For peer review only

For peer review only - http://bmjopen.bmj.com/site/about/guidelines.xhtml

TCSQ, SRSS, and the physical violence subscale. The mean score for PTSD symptoms on the PCL-C was 40.60 (SD = 15.01). As expected, the level of PTSD symptoms was negatively correlated with respondents' scores on the SSRS ($r = -0.188$, $P < 0.001$) and positive coping subscale of the TCSQ ($r = -0.164$, $P = 0.002$), respectively. Physical violence was positively associated with PTSD symptoms ($r = 0.259$, $P < 0.001$) .The level of PTSD symptoms was positively correlated with victims' scores on the negative coping subscale of theTCSQ ($r = 0.188$, $P < 0.001$).

Hierarchical Regression Analysis of Factors Related to PTSD Symptoms

The results of the hierarchical regression analysis are presented in Table 5.Variables that had a statistically significant association with PTSD were used as control variables. Gender had a significant effect on PTSD symptoms in the model (Block 1). As shown in Block 2, physical violence was positively associated with PTSD symptoms ($\beta = 1.216$, $P < 0.001$). As shown in Block 3, positive coping as measured by the TCSQ was negatively associated with PTSD symptoms ($\beta = -0.327$, $P = 0.002$), whereas, negative coping was positively associated with PTSD symptoms in the regression model ($\beta = 0.353$, $P = 0.001$). Furthermore, gender had a significant effect on PTSD symptoms, and men were more vulnerable to PTSD symptoms than women (Table 1). Therefore, we explored the potential correlates of PTSD symptoms in men and women (Table 6). As shown in Block 3,among the women, positive coping as measured by the TCSQ was significantly associated with PTSD symptoms ($\beta = -0.376$, $P = 0.001$), but the effect of positive coping was not significant in men.

Table 5. Hierarchical regression for exploring the correlates of PTSD symptoms.

Variables	Block 1 (β)	Block 2 (β)	Block 3 (β)	Block 4 (β)
Gender	-4.663^{*}	-3.282	-3.060	-3.012
Marital status	-2.021	-1.859	-2.626	-2.798
Occupation	-1.274	-1.918	-2.494	-2.414
Physical violence		1.216^{**}	1.015^{**}	1.028^{**}
SSRS			-0.193^{*}	-0.192^{*}
Positive coping of TCSQ			-0.327^{**}	-0.325^{**}

For peer review only - http://bmjopen.bmj.com/site/about/guidelines.xhtml

Negative coping of TCSQ			0.353**	0.361**
Extraversion				-0.049
Psychoticism				0.081
Neuroticism				0.042
F	5.189**	9.886**	10.544**	11.584**
R^2	0.041	0.098	0.170	0.246
ΔR^2	0.041	0.057**	0.072**	0.076*

Note. PTSD = post-traumatic stress disorder; SSRS = Social Support Rating Scale; TCSQ = Trait Coping Style Questionnaire.

*P<0.05, **P<0.01

For peer review only

Table 6. Hierarchical regression for exploring the correlates of PTSD symptoms in men and women, respectively.

	Variables	Mean (SD)	Block 1 (β)	Block 2 (β)	Block 3 (β)
Male					
n = 148	Physical violence	3.60 (3.16)	1.216**	1.033*	1.073**
	SSRS	41.25 (9.32)		-0.255	-0.257
	Positive coping of TCSQ	30.75 (7.28)		-0.298	-0.318
	Negative coping of TCSQ	27.21 (6.61)		0.467*	0.479*
	Extraversion	49.18 (10.04)			0.062
	Psychoticism	50.02 (8.62)			-0.282
	Neuroticism	49.99 (10.59)			-0.072
	F		8.727**	5.961**	4.182**
	R^2		0.056	0.143	0.173
	ΔR^2		0.056**	0.087**	0.030
Female					
n = 220	Physical violence	2.73 (2.83)	1.169**	0.955**	0.953**
	SSRS	42.05 (7.79)		-0.057	-0.066
	Positive coping of TCSQ	29.58 (7.16)		-0.376**	-0.376**
	Negative coping of TCSQ	26.72 (7.78)		0.296*	0.297*
	Extraversion	50.03 (10.54)			-0.121
	Psychoticism	50.14 (10.56)			0.006
	Neuroticism	50.12 (10.20)			-0.005
	F		13.441**	7.488**	4.557**
	R^2		0.058	0.122	0.131
	ΔR^2		0.058**	0.064**	0.009

Note. SD = standard deviations; PTSD = post-traumatic stress disorder; SSRS = Social Support Rating Scale; TCSQ = Trait Coping Style Questionnaire.

*P<0.05, **P<0.01

DISCUSSION

In this cross-sectional hospital-based study of healthcare workers exposed to physical violence, we

For peer review only - http://bmjopen.bmj.com/site/about/guidelines.xhtml

assessed the prevalence and correlates of PTSD symptoms. Our study found that the prevalence of physical violence among the healthcare workers was approximately 13.6% in the previous year. The results of a study conducted during 2009-2010 in Italy found that 13.4% of nurses were exposed to physical violence,[22] which is similar to the frequency found in this study. However, other studies have reported higher prevalence rates of physical violence than the current study.[23-24] The inconsistency in these findings may be attributed to cultural differences between countries or missing reports. PTSD was reported by 28.0% of the victims based on the scoring instructions of the PCL-C (i.e., 28.0% scored 50 points and above). We selected the PCL-C score of 50 and above as the standard cut-off due to the influence of traditional Chinese culture on the frequency of healthcare workers' encounters with traumatic events and the DSM IV-TR criteria for PTSD.[2] Previous studies have provided valuable information regarding the prevalence of PTSD among doctors and nurses.[28-31]The prevalence of PTSD among the healthcare workers exposed physical violence in our study was similar to that reported in Atlanta.[55] However, the prevalence rates of PTSD in these studies were different from the present study,[56-57] which might be attributed to differences in the studies' sample characteristics, designs, definitions, and diagnostic criteria for PTSD, due to their varied cultural backgrounds. Moreover, the prevalence of PTSD symptoms in our sample was higher than that of the general population (8%) in the USA.[58]This finding might be attributed to the fact that the general population's frequency of exposure to serious traumatic events is lower than that of healthcare workers. Similarly, nurses who work in intensive care units experience traumatic events more often than other healthcare workers do.[29]

Our study found that 21.2% of the victims of physical violence were at risk for developing PTSD and 28.0% met the full diagnostic criteria for PTSD. This finding suggests that physical violence had a strong influence on the mental health of healthcare workers. Approximately 53.0% (195/368) of the

For peer review only - http://bmjopen.bmj.com/site/about/guidelines.xhtml

victims reported having at least one PTSD criterion. The most commonly observed PTSD symptoms was re-experiencing (45.1%), followed by hyper-arousal (37.8%), and then avoidance (35.1%). A previous study also reported that healthcare workers in an emergency department were victims of direct workplace violence because they reported re-experiencing the violent event, followed by hyper-arousal and avoidance.[28] Laposa and Alden reported that re-experiencing an incident of physical violence was significantly and negatively associated with emergency department workers' ability to accomplish their work.[28] It is possible that the prevalence of symptoms of hyper-arousal and avoidance were not higher due to the distinctive characteristics of the healthcare workers' jobs and the hospital's culture, which required them to be able to shift their focus quickly and constantly. Healthcare workers who escaped slight injury during an episode of physical violence had to shift their focus rapidly to another patient after the event, and they could not avoid the work environment.[28]

As shown in the results of the Pearson's correlations and the hierarchical regression analysis, social support had a significant negative association with PTSD symptoms, and this finding is consistent with other research.[9 36 52 53] The level of PTSD symptoms was significantly and negatively correlated with the healthcare workers' scores for objective support and utilization of support. A previous study found that the Deterioration Model of Social Support has been useful in discriminating the potential of stressors to reduce support.[58]They found that disaster-induced erosion of perceived social support increased symptoms of depression among both primary and secondary victims; the loss of perceived social support also mediated psychological consequences.[59] The Deterioration Deterrence Model of Social Support which is similar to support-mobilization models, has been used to explain how the perceived deterioration of social support can be counteracted by higher levels of received social support.[59-60] If post-disaster support mobilization is implemented, stress should be positively

For peer review only - http://bmjopen.bmj.com/site/about/guidelines.xhtml

correlated with received support. At the same time, received support should be positively related to perceived support. Therefore, the receipt of support should suppress a negative relationship between stress and perceived support.[59-60] Victims of physical violence should be encouraged not to abandon their daily social activities because these activities have many important functions (e.g., they help people understand the needs of network members and inspire their participation in helping).[60] Daily contact is the most natural forum for sharing experiences, which might suppress negative emotions, provide opportunities for social comparison, and maintain a sense of friendship and feelings of being accepted.[60] It is important to recognize that stress caused by violence is persistent. Yet, a supportive hospital environment can help individuals cope with a wide range of stressful events and serve as a buffer against their negative health effects.[59-60]

Another significant effect of coping styles on PTSD symptoms was found in the present study. This result indicated that when healthcare workers encountered a traumatic event, a negative coping style was more likely to increase their proneness to developing PTSD symptoms. This finding is consistent with the results of other studies.[36 50 53] Positive coping was beneficial in preventing or alleviating PTSD symptoms in our study. In contrast, a previous investigation found that activing coping had a positive relationship with PTSD.[36] Unexpectedly, the three personality factors were not significantly associated with PTSD symptoms. However, Lawrence and Fauerbach's study found that individuals with higher Neuroticism scores exhibited more PTSD symptoms.[36]

An important finding of the present study was revealed in the univariate analyses. We found that the men exposed to traumatic events were more likely to exhibit PTSD symptoms than the women were. This result was different from the findings reported in earlier studies that women are more likely to develop PTSD symptoms.[6 17 19]This finding might be attributed to gender differences in responses to

For peer review only - http://bmjopen.bmj.com/site/about/guidelines.xhtml

different traumatic events and in social networks.[61-62] This phenomenon also might be attributable to the fact that the injuries sustained by the men after experiencing physical violence were more severe than those of the women. After experiencing physical violence, the women were likely to receive more social support than the men suggesting that women were more often regarded as a vulnerable group.

These findings suggest that social support, exposure to physical violence, and coping styles are closely related to PTSD symptoms. Therefore, we recommend interventions based on the social cognitive theory.[63] For example, social support has been found to be an important protective factor in reducing stress and depression, and improving health.[63] After the occurrence of a traumatic event, enabling function of social support can enhance self-efficacy, thereby promoting recovery from the trauma.[63]

The present study has several limitations. First, we used the PCL-C to assess PTSD symptoms rather than a standard clinical diagnostic method. Consequently, the prevalence of PTSD might be overestimated. Second, the study's findings need to be replicated in a longitudinal study. Finally, our results are specific to Chinese healthcare workers exposed to physical violence in the past 12 months; thus, the low representativeness of the sample due to the use convenience sampling limits the generaliz ability of the results. The inclusion of healthcare workers from a wider range of careers in a more representative sample should contribute to the ability to generalize the results of future studies.

CONCLUSIONS

The prevalence of PTSD among healthcare workers exposed physical violence was similar to that in Atlanta. The positive effects of social support on PTSD symptoms suggest that social support has practical implications for psychological interventions to promote mental health. Furthermore, the

For peer review only - http://bmjopen.bmj.com/site/about/guidelines.xhtml

For peer review only

coping styles of the healthcare workers in this study influenced the development of PTSD symptoms. Therefore, it is imperative to use positive coping methods and to receive social support after experiencing a traumatic event.

Acknowledgments

The authors thank all the healthcare workers, managers and Chinese Hospital Association for their assistance and support for this project.

Author Contributions

LS and LF designed the study. LS, LW, XJ, BP and LF collected data. ZL, LW, XJ,HM, XL and AL analysed the data. LS and LF drafted the manuscript. LS, ZL and LF revised the manuscript.

Funding

This study was funded by the National Natural Science Foundation of China, grant number 71473063.

Competing interests None declared.

Data sharing statement No additional data are available.

REFERENCES

1. Guo W, Xue JM, Shao D, *et al.* Effect of the interplay between trauma severity and trait neuroticism on posttraumatic stress disorder symptoms among adolescents exposed to a pipeline explosion. *PLoS One* 2015;10(3):e0120493.
2. Association AP. Diagnostic and statistical manual of mental disorders fourth edition, text revision (DSM-IV-TR). 2000.
3. Andreasen NC. What is post-traumatic stress disorder? *Dialogues Clin Neurosci* 2011;13:240–243.

For peer review only - http://bmjopen.bmj.com/site/about/guidelines.xhtml

4. Association AP. Diagnostic and statistical manual of mental disorders fifth edition, text revision (DSM-V). 2013.

5. Van Der, Kolk BA.The psychobiology and psychopharmacology of PTSD.*Hum Psychopharmacol* 2001;16(S1):S49 – S64.

6. Shalev AY. Post-traumatic stress disorder. Disorder takes away human dignity and character. *BMJ* 2001;322(7297):1301–1304.

7. Hu BS, Liang YX. Post-traumatic stress disorder. *J Occup Environ Hyg* 1997;(5):266-269.

8. Klein S, Alexander DA. Epidemiology and presentation of post-traumatic disorders. *Psychiatry* 2009;8(8):282-287.

9. Fan F, Zhang Y, Yang Y, *et al.* Symptoms of posttraumatic stress disorder, depression, and anxiety among adolescents following the 2008 Wenchuan earthquake in China. *J Trauma Stress* 2011;24(1): 44–53.

10. Boe HJ, Holgersen KH, Holen A. Mental health outcomes and predictors of chronic disorders after the North Sea oil rig disaster: 27-year longitudinal follow-up study. *J Nerv Ment Dis* 2011;199(1):49–54.

11. Wang YL, Xie W, Yang ZH, *et al.* Research progress of posttraumatic stress disorder in China. *Chinese Journal of Applied Psychology* 2005;11(2):176-180.

12. Alexander DA, Klein S. Ambulance personnel and critical incidents: impact of accident and emergency work on mental health and emotional well-being. *Brit J Psychia* 2001;178(1):76.

13. Jonsson A, Segesten K, Mattsson B. Post-traumatic stress among Swedish ambulance personnel. *Emerg Med J* 2003;20(1):79-84.

14. Laposa JM, Alden LE, Fullerton LM. Work stress and posttraumatic stress disorder in ED nurses

For peer review only - http://bmjopen.bmj.com/site/about/guidelines.xhtml

/personnel. *J Emerg Nurs* 2003;29(1):23-28.

15. Shoji K, Lesnierowska M, Smoktunowicz E, *et al*. What Comes First, Job Burnout or Secondary Traumatic Stress? Findings from Two Longitudinal Studies from the U.S. and Poland. *Plos One* 2015;10(8):e0136730.

16. Boer JD, Lok A, Verlaat EV, *et al*. Work-related critical incidents in hospital-based health care providers and the risk of post-traumatic stress symptoms, anxiety, and depression: A meta-analysis. *Soc Sci Med* 2011;73(2):316-326.

17. Krug EG, Mercy JA, Dahlberg LL, *et al*. The world report on violence and health. *Lancet* 2002; 360(9339):1083.

18. Ray MM. The dark side of the job: Violence in the emergency department. *J Emerg Nurs* 2007;33 :257-261.

19. Ahmad M, Al-Rimawi R, Masadeh A, *et al.* Workplace Violence by Patients and their Families against Nurses: Literature Review. *Int J NursTermin Cl* 2015;2(4):46-55.

20. Ramacciati N, Ceccagnoli A, Addey B. Violence against nurses in the triage area: An Italian qualitative study. *Int Emerg Nurs* 2015;23(4):274-280.

21. Harrell, Erika. Workplace Violence, 1993-2009: National Crime Victimization Survey and the Census of Fatal Occupational Injuries. *Digitalcommons* 2011.

22. Magnavita N, Heponiemi T. Workplace violence against nursing students and nurses: an Italian experience. *J Nurs Scholarsh* 2011;43(2):203-210.

23. Fute M, Mengesha ZB, Wakgari N, *et al*. High prevalence of workplace violence among nurses working at public health facilities in Southern Ethiopia. *BMC Nurs* 2015;14:9.

24. Al-Omari H. Physical and verbal workplace violence against nurses in Jordan. *Int Nurs Rev*

For peer review only - http://bmjopen.bmj.com/site/about/guidelines.xhtml

2015;62(1):111–118.

25. Schablon A, Zeh A, Wendeler D, *et al.* Frequency and consequences of violence and aggression towards employees in the German healthcare and welfare system: a cross-sectional study. *BMJ Open* 2011;2(5):421-421.

26. Najafi F, Fallahi-Khoshknab M, Dalvandi A, *et al.* Workplace violence against Iranian nurses: A systematic review. *J Health Promot Manage* 2014;3:72-85.

27. Pan Y, Yang XH, He JP, *et al*. To be or not to be a doctor, that is the question: a review of serious incidents of violence against doctors in China from 2003–2013. *J public health* 2015;23(2):111 -116

28. Laposa JM, Alden LE. Posttraumatic stress disorder in the emergency room: exploration of a cognitive model.*Behav Res Ther* 2003;41(1):49-65.

29. Mills LD, Mills TJ. Symptoms of post-traumatic stress disorder among emergency medicine residents. *J Emerg Med* 2005;28(1):1–4.

30. Friedman, MJ, Resick, PA, Bryant, R.A, *et al.* Considering PTSD for DSM-5. *Depress Anxiety* 2011;28(9):750-769.

31. Robertson N, Perry A. Institutionally based health care workers' exposure to traumatogenic events: Systematic review of PTSD presentation. *Journal of Traumatic Stress* 2010;23(3):417-420.

32. Kornblith AB, Herndon JE, Weiss RB, *et al.* Long-term adjustment of survivors of early-stage breast carcinoma, 20 years after adjuvant chemotherapy. *Cancer* 2003;98(4):679–689.

33. Rourke MT, Hobbie WL, Schwartz L, *et al.* Posttrauamatic stress disorder (PTSD) in young adult survivors of childhood cancer. *Pediatr Blood Cancer* 2007; 49(2): 177–82.

For peer review only - http://bmjopen.bmj.com/site/about/guidelines.xhtml

34. Perrin M, Vandeleur C L, Castelao E, *et al*. Determinants of the development of post-traumatic stress disorder, in the general population. *Soc Psych Psych Epid* 2014;49(3):447-457.

35. Breslau N, Davis G C, Andreski P, *et al*. Traumatic events and posttraumatic stress disorder in an urban population of young adults. *Arch Gen Psychiatry* 1991;48(3):216-222.

36. Lawrence JW, Fauerbach JA. Personality, coping, chronic stress, social support and PTSD symptoms among adult burn survivors: a path analysis. *J Burn Care Rehabil* 2003;24(1):63.

37. Kerasiotis B, Motta RW. Assessment of PTSD symptoms in emergency room, intensive care unit, and general floor nurses. *Int J Emerg Ment Health* 2004;6(3):121-133.

38. Boer JD, Lok A, Verlaat EV, *et al*. Work-related critical incidents in hospital-based health care providers and the risk of post-traumatic stress symptoms, anxiety, and depression: A meta-analys is. *Soc Scie Med* 2011;73(2):316-326.

39. Shoji K, Lesnierowska M, Smoktunowicz E, *et al*. What Comes First, Job Burnout or Secondary Traumatic Stress? Findings from Two Longitudinal Studies from the U.S. and Poland. *Plos One* 2015;10(8):e0136730.

40. Cieslak R, Anderson V, Bock J, *et al*. Secondary traumatic stress among mental health providers working with the military: Prevalence and its work- and exposure-related correlates.*J Nerv Ment Dis* 2013;201(11):917-25.

41. ILO/ICN/WHO/PSI. Framework Guidelines for Addressing Workplace Violence in the Health Sector. Geneva: ILO/ICN/WHO/PSI Joint Programme on Workplace Violence in the Health Sector; 2002.

42. Zampieron A, Galeazzo M, Turra S, *et al.* Perceived aggression towards nurses: study in two Italian health institutions. *J Clin Nurs* 2010;19:2329–41.

For peer review only - http://bmjopen.bmj.com/site/about/guidelines.xhtml

For peer review only

43. Battles ED. An exploration of post-traumatic stress disorder in emergency nurses following Hurricane Katrina. *J Emerg Nurs* 2007;33(4):314-318.

44. Dirk Richter K B. Post-traumatic stress disorder following patient assaults among staff members of mental health hospitals: a prospective longitudinal study. *BMC Psychiatry* 2006;6(1):15.

45. Weathers FW, Litz BT, Herman DS, *et al*. The PTSD checklist: reliability, validity & diagnostic utility. Paper presented at the Annual Meeting of the International Society for Traumatic Stress Studies, *San Antonio, TX* 1993.

46. Wu Z, Xu J, Sui Y. Posttraumatic stress disorder and posttraumatic growth coexistence and the risk factors in Wenchuan earthquake survivors. *Psychiatry Res* 2016; 237:49-54.

47. Qian M, Wu G, Zhu R, *et al.* Development of the revised Eysenck Personality Questionnaire Short Scale for Chinese (EPQ-RSC). *Acta Psychologica Sinica* 2000;32(3):317-323.

48. Zhang Z, Shi Z, Wang L, *et al.* Post-traumatic stress disorder, anxiety and depression among the elderly: a survey of the hard-hit areas a year after the Wenchuan earthquake. *Stress Health* 2012; 28(1):61-68.

49. Cui Y, Tian SS, Qiao N, *et al.* Associations of Individual-Related and Job-Related Risk Factors with Nonfatal Occupational Injury in the Coal Workers of Shanxi Province: A Cross-Sectional Study. *PLoS One* 2015;10(7):e0134367.

50. Zhang L, Zhang J X, Wen-Juan X U, *et al.* An evaluation of validity and reliability on the trait coping style questionnaire(TCSQ) using for Chinese soldier. *Chinese Journal of Disease Control & Prevention*2010.

51. Li-Na L I, Zhao Y, Cui X J, et al. The Influence Factors and Status on Male Vicarious Trauma of Disaster. *Chinese General Practice* 2014.

For peer review only - http://bmjopen.bmj.com/site/about/guidelines.xhtml

52. Feng S, Tan H, Benjamin A, *et al*. Social support and posttraumatic stress disorder among flood victims in Hunan, China. *Ann Epidemio*l. 2007;17(10):827-833.

53. Li H, Yan C, Zhu S, *et al.* Correlation among coping style, social support, and negative emotion in infertile women. Zhong nan da xue xue bao. Yi xue ban=Journal of Central South University. *Medical sciences* 2011;36(2):138-142.

54. Chang-Fei LU, Jia CX, Zhang JY, *et al.* Reliability and validity of Social Support Rating Scale in rural suicides. *Chinese Mental Health Journal* 2011;25(3):218-222.

55. Mealer ML, Shelton A, Berg B, *et al*. Increased prevalence of post-traumatic stress disorder symptoms in critical care nurses. *Am J Resp Crit Care* 2007;175(175):693-697.

56. Zafar W, Khan UR, Siddiqui SA, *et al*. Workplace Violence and Self-reported Psychological Health: Coping with Post-traumatic Stress, Mental Distress, and Burnout among Physicians Working in the Emergency Departments Compared to Other Specialties in Pakistan.*J Emerg Med* 2015;50(1):167-177.

57. Johansen VA, Eilertsen DE, Nordanger D, *et al.* Prevalence, comorbidity and stability of post-traumatic stress disorder and anxiety and depression symptoms after exposure to physical assault: an 8-year prospective longitudinal study. *Nord J Psychiatry* 2013; 67(1):69-80.

58. BreslauN. The epidemiology of post-traumatic stress disorder; what is the extent of the problem? *Journal of Clinical Psychiatric* 2001;62:16-22.

59. Kaniasty K, Norris FH. Mobilization and deterioration of social support following natural disasters. *Current Directions in Psychological Science* 1995;4(3):94-98.

60. Norris FH, Kaniasty K. Received and perceived social support in times of stress: a test of the social support deterioration deterrence model. *Journal of Personality & Social Psychology*

For peer review only - http://bmjopen.bmj.com/site/about/guidelines.xhtml

1996;71(3):498-511.

61. Freedman SA, Gluck N, Tuval-Mashiach R, *et al*. Gender differences in responses to traumatic events: a prospective study. *J Trauma Stress* 2002;15:407-413.

62. Pratchett LC, Pelcovitz MR, Yehuda R. Trauma and violence: are women the weaker sex? *Psychiatr Clin North Am* 2010; 33: 465–474.

63. Benight CC, Bandura A. Social cognitive theory of posttraumatic recovery: the role of perceived self-efficacy. *Behaviour Research & Therapy* 2004;42(10):1129.

For peer review only

For peer review only - http://bmjopen.bmj.com/site/about/guidelines.xhtml

STROBE 2007 (v4) Statement—Checklist of items that should be included in reports of *cross-sectional studies*

Section/Topic	Item #	Recommendation	Reported on page #
**Title and abstract**	1	(*a*) Indicate the study's design with a commonly used term in the title or the abstract	Page 1, line 6; p. 2 , line 21
		(*b*) Provide in the abstract an informative and balanced summary of what was done and what was found	P.2 ; p.3, line 6-17
**Introduction**			
Background/rationale	2	Explain the scientific background and rationale for the investigation being reported	P.4; p.5; p.6, line 4-32
Objectives	3	State specific objectives, including any prespecified hypotheses	P.6, line 34-39
**Methods**			
Study design	4	Present key elements of study design early in the paper	P.7, line 14-24
Setting	5	Describe the setting, locations, and relevant dates, including periods of recruitment, exposure, follow-up, and data collection	P.6, line 47-58; p.7, line 4-14; line 24-39
Participants	6	(*a*) Give the eligibility criteria, and the sources and methods of selection of participants	P.7, line 42-57; p.8, 4
Variables	7	Clearly define all outcomes, exposures, predictors, potential confounders, and effect modifiers. Give diagnostic criteria, if applicable	P.8, line 11-29
Data sources/	8*	For each variable of interest, give sources of data and details of methods of assessment (measurement). Describe	P.8, line 31-57; p.9;

For peer review only

measurement		comparability of assessment methods if there is more than one group	p.10; p. 11, line 4-39
Bias	9	Describe any efforts to address potential sources of bias	P.6, line 57; p.7,line 4
Study size	10	Explain how the study size was arrived at	P.7, line 24-32
Quantitative variables	11	Explain how quantitative variables were handled in the analyses. If applicable, describe which groupings were chosen and why	p.11, line 52-17; p.12, line 4-6; P.6, line 11-31
Statistical methods	12	(*a*) Describe all statistical methods, including those used to control for confounding	P.11, line 52-57; p. 12, line 2-24
		(*b*) Describe any methods used to examine subgroups and interactions	P.12, line 4-16
		(*c*) Explain how missing data were addressed	P.11, line 47-52
		(*d*) If applicable, describe analytical methods taking account of sampling strategy	P.12, line 4-16
		(*e*) Describe any sensitivity analyses	P.11, line 54; p.12, line 16-24
**Results**			
Participants	13*	(a) Report numbers of individuals at each stage of study—eg numbers potentially eligible, examined for eligibility, confirmed eligible, included in the study, completing follow-up, and analysed	P.13, line 42-47
		(b) Give reasons for non-participation at each stage	P.7, line 47-57
		(c) Consider use of a flow diagram	No
Descriptive data	14*	(a) Give characteristics of study participants (eg demographic, clinical, social) and information on exposures and potential confounders	P.13; p.14

For peer review only

		(b) Indicate number of participants with missing data for each variable of interest	P.11, line 47-49
Outcome data	15*	Report numbers of outcome events or summary measures	P.15, line 4-50
Main results	16	(*a*) Give unadjusted estimates and, if applicable, confounder-adjusted estimates and their precision (eg, 95% confidence interval). Make clear which confounders were adjusted for and why they were included	P.17, line 17-58; p.18, line 3-47
		(*b*) Report category boundaries when continuous variables were categorized	P.8, line 9-29
		(*c*) If relevant, consider translating estimates of relative risk into absolute risk for a meaningful time period	P.13, line42-59; p.14; p.16
Other analyses	17	Report other analyses done—eg analyses of subgroups and interactions, and sensitivity analyses	P.17, line 16-44
**Discussion**			
Key results	18	Summarise key results with reference to study objectives	P.18, line 54-57; p.19. Line 4-9
Limitations	19	Discuss limitations of the study, taking into account sources of potential bias or imprecision. Discuss both direction and magnitude of any potential bias	P. 22, Line 27-42
Interpretation	20	Give a cautious overall interpretation of results considering objectives, limitations, multiplicity of analyses, results from similar studies, and other relevant evidence	P.19; p.20; p.21; p.22, line4-42
Generalisability	21	Discuss the generalisability (external validity) of the study results	P.22, line 12-42
**Other information**			
Funding	22	Give the source of funding and the role of the funders for the present study and, if applicable, for the original study on which the present article is based	P.23, line 27-29

*Give information separately for cases and controls in case-control studies and, if applicable, for exposed and unexposed groups in cohort and cross-sectional studies.

For peer review only

Note: An Explanation and Elaboration article discusses each checklist item and gives methodological background and published examples of transparent reporting. The STROBE checklist is best used in conjunction with this article (freely available on the Web sites of PLoS Medicine at http://www.plosmedicine.org/, Annals of Internal Medicine at http://www.annals.org/, and Epidemiology at http://www.epidem.com/). Information on the STROBE Initiative is available at www.strobe-statement.org.

For peer review only

BMJ Open

Prevalence and correlates of symptoms of post–traumatic stress disorder among Chinese healthcare workers exposed to physical violence: a cross–sectional study

Journal:	*BMJ Open*
Manuscript ID	bmjopen-2017-016810.R2
Article Type:	Research
Date Submitted by the Author:	25-Jun-2017
Complete List of Authors:	Shi, Lei<br>Wang, Lingling ; Chinese Hospital Association, Department of Autonomous Protection<br>Jia, Xiaoli<br>Li, Zhe<br>Mu, Huitong<br>Liu, Xin<br>Peng, Boshi<br>Li, Anqi<br>Fan, Lihua; Harbin Medical University, Department of Health Management , School of Public Health
**Primary Subject Heading**:	Health policy
Secondary Subject Heading:	Health policy, Health services research, Mental health
Keywords:	Posttraumatic stress disorder symptoms (PTSD), physical violence, social support, coping styles, MENTAL HEALTH, personality

SCHOLARONE™
Manuscripts

Prevalence and correlates of symptoms of post–traumatic stress disorder among Chinese healthcare workers exposed to physical violence: a cross–sectional study

Lei Shi,[1] Lingling Wang,[2] Xiaoli Jia,[2] Zhe Li,[1] Huitong Mu,[1] Xin Liu,[1] Boshi Peng,[3] Anqi Li,[1] Lihua Fan[1]

Correspondence to: Lihua Fan, School of Public Health, Harbin Medical University, 157 Baojian Road, Nangang District, Harbin 150081, China; E-mail: lihuafan@126.com

[1]Department of Health Management, School of Public Health, Harbin Medical University, Harbin, Heilongjiang, China

[2]Department of Autonomous Protection, Chinese Hospital Association, Beijing, China

[3] Department of Scientific Research, The Second Affiliated Hospital of Harbin Medical University, Harbin, Heilongjiang, China

For peer review only

For peer review only - http://bmjopen.bmj.com/site/about/guidelines.xhtml

Abstract

Objectives

Post–traumatic stress disorder (PTSD) is a common psychological maladjustment to undergoing a traumatic event. Our aim was to measure the prevalence of PTSD among Chinese healthcare workers exposed to physical violence, and explore the associations of their demographic characteristics, social support, personality traits, and coping styles with their PTSD symptoms.

Methods

A cross-sectional study was conducted using the: Workplace Violence Scale, Posttraumatic Stress Disorder Checklist–Civilian Version (PCL–C), Social Support Rating Scale (SSRS), Revised Eysenck Personality Questionnaire–Short Scale and Trait Coping Style Questionnaire. We used convenience sampling method to collect data from March 2015 to September 2016.Healthcare workers (N = 2,706) from 39 public hospitals located in Heilongjiang, Hebei, and Beijing Provinces of China completed the questionnaires (effective response rate = 84.25%).

Results

Overall, the prevalence of physical violence in the previous 12 months was 13.60% (N = 2,706).The prevalence of PTSD among the healthcare workers who experienced physical violence was 28.0% (n = 368). Most of the victims of physical violence (50.80%) did not exhibit PTSD symptoms based on their PCL–C scores, and 47.0% did not manifest the diagnostic criteria for PTSD after experiencing physical violence. The level of PTSD symptoms was negatively correlated with their scores on the SSRS ($r = -0.188$, $P < 0.001$). The hierarchical regression analysis (Block 3) revealed that in women, positive coping was significantly associated with PTSD symptoms ($\beta = -0.376$, $P = 0.001$). However, the effect of positive coping was not significant in men.

For peer review only - http://bmjopen.bmj.com/site/about/guidelines.xhtml

For peer review only

Conclusions

The results suggest that the aftermath of physical violence contributes to current prevalence of PTSD. The positive effects of social support on PTSD symptoms suggest that it has practical implications for interventions to promote psychological health. The healthcare workers' coping styles influenced the development of PTSD symptoms. Therefore, adopting effective coping styles and receiving social support have potential roles in the recovery from trauma after experiencing physical violence.

Strengths and limitations of this study

▪ In China, few studies have been conducted on PTSD symptoms following healthcare workers' exposure to physical violence.

▪ We assessed the prevalence of PTSD and explored the correlates of PTSD symptoms among Chinese healthcare workers exposed to physical violence.

▪ Our study was conducted at 39 public hospitals in three provinces using convenience sampling. Therefore, the representativeness of the sample is limited.

▪ The retrospective approach to collecting data using self-reports of PTSD symptoms might have led to recall and report bias.

For peer review only - http://bmjopen.bmj.com/site/about/guidelines.xhtml

BACKGROUND

Post–traumatic stress disorder (PTSD) is a psychological state of imbalance, characterized by a series of chronic emotional reactions to a traumatic event, including re–experiencing, avoidance, and heightened arousal, as outlined in the Diagnostic and Statistical Manual of Psychiatric Disorders–4th edition (DSM–IV).[1-3] However, the criteria for PTSD in the manual's fifth edition (DSM–5) include not three but four symptom clusters: re–experiencing, avoidance, negative alterations in mood and cognition, and hyperarousal.[4] It is worth noting that PTSD has shifted from its classification as an anxiety disorder in the DSM–IV to a new category of Trauma and Stress–related Disorders in the DSM–5.[4] Although a substantial number of studies indicate that almost all people exhibit intrusive and repetitious symptoms after exposure to excessive stress,[5] only a small percentage develop avoidance and hyper–arousal symptoms. Most individuals showing PTSD symptoms after exposure to a traumatic event recover within weeks or months. However, 10%–25% might develop chronic PTSD that lasts for several months or years, or even a lifetime.[6]

PTSD originated from reports of the war–related trauma, and was applied gradually to a variety of man–made and natural disasters.[7] Scholars have reported that the incidence of PTSD among male and female Vietnam veterans in the USA is 15.2% and 8.5%, respectively.[8] Moreover, most of the Chinese studies on PTSD have focused on wars, traffic accidents, and natural disasters.[9-10] Differences in the incidence rates of PTSD for different types of trauma have been reported in China. For instance, the prevalence of PTSD has been reported to be 8.65% among soldiers assigned to military vehicles at high altitudes, 33.89% among flood–disaster survivors, 18.8% among earthquake survivors, 41% among traffic–accident survivors, and 78.6% among survivors of a serious explosions.[11]

PTSD symptoms and the full range of criteria comprising a PTSD diagnosis have been observed

For peer review only - http://bmjopen.bmj.com/site/about/guidelines.xhtml

in rescue and ambulance personnel.[12-13] Healthcare workers typically are exposed to two types of trauma in the hospital setting: direct (personal involvement in traumatic events through confrontations resulting in their own traumatic experiences e.g., workplace violence) and indirect (non–personal involvement in traumatic events through others' confrontations resulting in other people's traumatic experiences e.g., witnessing other people's direct experiences of workplace violence, caring for dying patients, and threats of severe injury or exposure to trauma).[4 14-16] In the present study, a traumatic event refers to a healthcare worker's exposure to physical violence in the workplace. Workplace violence is divided into physical and psychological violence.[17] Physical violence causes more serious physical and psychological damage (e.g., PTSD, anxiety, fear, and depression) than other forms of violence.[18-20] Physical violence refers to the use of physical force against an individual or a group, and can lead to physical, psychological, or sexual harm; it includes hitting, shooting, kicking, slapping, pushing, biting, pinching, wounding using sharp objects, and sexual assault and rape.[17] Approximately 50% of healthcare workers have experienced at least one violent incident during their working lives.[21] During the past 12 months, the incidence rate of physical violence for nurses in different countries has ranged from 9.1% to 56.0%.[22-25] The results of a systematic review of studies conducted in Iran indicated that the most common types of physical violence experienced by 43% of participants were pushing or pinching.[26] In China, physician–patient conflicts present a growing trend, with an increase in the number of healthcare workers killed by patients or their relatives to 24, and an increase in injures from 2003 to 2013.[27] Several studies have estimated the prevalence of PTSD among emergency department staff to range from 10% to 25%.[28-30] Noelle Robertson and Andrew Perry conducted a systematic review of PTSD research investigations; the results showed that the prevalence of PTSD ranged from 8% to 29% among different hospital–based departments.[31] There are also reports of the occurrence of

For peer review only - http://bmjopen.bmj.com/site/about/guidelines.xhtml

PTSD among Chinese nurses working in emergency departments, intensive care units, and operating rooms. However, the number of research studies on PTSD among healthcare workers has been relatively few in China.

Demographic variables (e.g., age, gender, and educational level)and psychological and social variables (e.g., personality, coping style, and social support) have been found to be significantly associated with cancer–related PTSD symptoms.[32-33] Previous studies have found that the risk of PTSD was most strongly associated with neuroticism and problem–focused coping strategies in the general population.[34-35] Neuroticism was the most critical personality dimension in predicting PTSD, and avoidant coping and social support mediated the relationship between neuroticism and PTSD in a high proportion of adult–burn survivors.[36] Social support has been reported to play a significant role in helping nurses cope with work–related stress.[37] A meta-analysis indicated that work–related critical incidents were positively related to PTSD symptoms.[38]

In this study, we aimed to assess the prevalence of PTSD, and to explore the associations of demographic characteristics, social support, personality characteristics, and coping styles with PTSD symptoms among Chinese healthcare workers exposed to physical violence.

METHODS

Participants and Procedures

A cross–sectional study was conducted from March 2015 through September 2016 with a sample of healthcare workers employed by 39 public hospitals located in Heilongjiang, Hebei, and Beijing Provinces of China. The 39 public hospitals that served as the research settings were chosen using convenience sampling. All investigators were trained using a uniform survey manual before they began

For peer review only - http://bmjopen.bmj.com/site/about/guidelines.xhtml

to collect data. Qualified investigators were appointed to collect data. We obtained permission from the managers and the medical dispute resolution and human resources departments of the hospitals. The investigators conducted face–to–face surveys by using an anonymous, self–administered questionnaire. We purposely selected 3 public hospitals in Harbin (the First Affiliated Hospital of Harbin Medical University, the Second Affiliated Hospital of Harbin Medical University, and the Fourth Affiliated Hospital of Harbin Medical University) as the sites for our pilot study before the formal investigation. A total of 150 questionnaires were distributed and returned (these data were excluded from the main study). A total of 3,212 healthcare workers (i.e., physicians, nurses and medical technicians) were investigated using convenience sampling in the formal investigation. The researchers and hospital coordinators distributed and collected the questionnaires that were completed by the healthcare workers immediately. A total of 2,706 valid questionnaires were returned, and the effective response rate was 84.25%. This study's focus was only on PTSD symptoms among healthcare workers exposed to physical violence; thus, only 368 responses were considered valid data and were analyzed in the present study.

The inclusion criteria for participation in this study were as follows: (1) at least one year of work experience; (2) voluntary participation; (3) participation would not affect the participation's work; and (4) experience of physical violence in the previous 12 months. Individuals were excluded if they (1) had received any psychological treatment after experiencing physical violence; (2) experienced other traumatic events, including workplace psychological violence or serious life events (e.g., domestic violence or attacks by criminals), serious accidents (e.g., fires, explosions, or traffic accidents), natural disasters (e.g., typhoons, earthquakes, or floods), or (3) were indirectly exposed to trauma,[39-40] (e.g., witnessing other people experience traumatic events).

For peer review only - http://bmjopen.bmj.com/site/about/guidelines.xhtml

Questionnaire

Demographic characteristics

Demographic data on the healthcare workers were collected, including gender, age, marital status, educational status, professional title, department, occupation, and work experience. Age was categorized as ≤ 30, 31–50, and ≥ 51 years old. Marital status was categorized as married and single/ divorced/widowed. Educational status was classified as junior college or below, undergraduate, and graduate. Occupation was divided into three groups: physician, nurse, and medical technician. Professional title was categorized as primary, intermediate, and senior. Department was classified as emergency department, internal medicine, surgery, obstetrics and gynecology, pediatrics, and other. Work experience was divided into four categories: ≤ 4, 5–10, 11–20, and ≥ 21 years.

Workplace Violence Scale

The Workplace Violence (WPV) Scale developed by the International Labour Office, International Council of Nurses, World Health Organization, and Public Services International Joint Programme on Workplace Violence in the Health Sector in 2003and the revised Survey of Violence Experienced by Staff were used to measure workplace violence.[41-42] We obtained permission to use these scales. The scale used in this study consists of 2 dimensions (physical violence and psychological violence) and has 9 items that were adopted from these scales. Each item is scored on a 4–point scale reflecting respondents' frequency of exposure to violence (0 = 0 times, 1 = 1 time, 2 = 2–3 times, and 3 = ≥ 4 times). The total possible score ranges from 0 to 27, with a higher total score indicating a higher frequency of exposure to WPV. The physical violence subscale consists of 6 items, thus, the total possible score ranges from 1 to 18. In the present study, Cronbach's α for the WPV Scale was 0.86.

PTSD

For peer review only - http://bmjopen.bmj.com/site/about/guidelines.xhtml

The PTSD Checklist–Civilian Version (PCL–C),which has been used to measure PTSD symptoms among healthcare workers was used in the present study.[43-44] It consists of 17 self–report items, which comprise three dimensions, namely, re–experiencing, avoidance/numbing and hyper–arousal. The three dimensions correspond to the DSM–IV symptoms criteria for PTSD.[2] The response options for each item on the PCL–C are rated from 1 (not at all) to 5 (extremely), based on the extent to which the respondent has been troubled by specific symptoms in the past month. The total possible score is calculated by adding the scores for all items, and it ranges from 17 to 85 points, with a higher score indicating a higher risk for PTSD symptoms. A total score ≥ 50 is indicative of the full PTSD diagnosis (sensitivity = 0.82; specificity = 0.83; kappa = 0.64).[45] In this study, the traumatic event in the original PCL–C was replaced by physical violence. The reliability and validity of this instrument have been shown to be high in a wide range of Chinese samples.[46] The present study revealed that Cronbach's α for the PCL–C was 0.934, and for the three subscales it was 0.872 (re–experiencing), 0.921 (avoidance), and 0.926 (hyper–arousal).

Revised Eysenck Personality Questionnaire–Short Scale

Personality traits were measured using the Revised Eysenck Personality Questionnaire–Short Scale for Chinese (EPQ–RSC).[47-48] The EPQ–RSC consists of 48 items, categorized into 4 subscales reflecting personality traits: Extraversion, Neuroticism, Psychoticism, and Lie. Each item is scored on a dichotomous scale (1=Yes, 0=No) to measure personality characteristics. The scores of the positively and negatively worded items are summed in accordance with each personality trait. Early studies found the EPQ–RSC to have high reliability and validity as a measure of personality traits inChina.[48 -49] The total score for the Extraversion subscale indicates introversion when it is less than 43.3, intermediate when it is from 43.3 to 56.7, and extraversion when it is greater than 56.7.[49] For the Psychoticism

For peer review only - http://bmjopen.bmj.com/site/about/guidelines.xhtml

subscale, tough-minded is defined as a total score greater than 56.7; intermediate is defined as a total score between 43.3 and 56.7, and mild is defined as a total score less than 43.3.[49] For the Neuroticism subscale, a total score of less than 43.3 defines emotional stability, whereas a total score from 43.3 to 56.7 defines intermediate, and a total score greater than 56.7 defines emotional instability.[49] For the Lie subscale, a total score of 60 or greater indicates that information provided by the respondent might beunreliable.[49] In this study, Cronbach's α for the EPQ–RSC was 0.903. The internal consistency coefficients were 0.854, 0.756, 0.791, and 0.762, for the Extraversion, Neuroticism, Psychoticism, and Lie subscales, respectively.

Trait Coping Style Questionnaire

The Trait Coping Style Questionnaire (TCSQ) was used in this study to assess participants' style of coping with life events.[37 50] The TCSQ consists of 20 items, including 10 items measuring positive coping and 10 items measuring negative coping. Positive coping refers to individuals who, when faced with a problem, tend to deal with it in a positive way, and are able to quickly forget unpleasant aspects. Negative coping refers to the tendency to use negative coping methods to deal with problems and vent frustrations to other people, which makes it is easier to ignore unpleasant thoughts. For example, when conflicts with others, arise, individuals who use negative coping will ignore the opposing side for a long time.[51] Each item is rated on a 5–point Likert scale. The total possible score for positive and negative coping is calculated by adding the scores for all the items. Previous studies have found the TCSQ to have high reliability and validity as a measure of coping style in China.[50-51] In this study, Cronbach's α for the total scale was 0.845, and the internal consistency coefficients of the subscales were α = 0.823 (positive coping), and α = 0.863 (negative coping).

Social Support Rating Scale

For peer review only - http://bmjopen.bmj.com/site/about/guidelines.xhtml

Social support was evaluated using the Chinese version of the Social Support Rating Scale (SSRS),[52-54] which is a brief measure of the overall situation of respondents' social support. This 10–item scale is divided into 3 dimensions: subjective support, objective support and utilization of support. Subjective support refers to an individual's emotional experience of being respected, supported, and understood by their social group, and it is closely related to the individual's subjective feelings. Objective support refers to visible support, including material and direct assistance, social networks, group relationships, and the individual's degree of participation in societal activities with family, friends, and colleagues (e.g., marriage). A low level of social support is defined as a total score between 12 and 44, an intermediate level as a total score between 45 and 54, and high level as a total score greater than 55.[54] The present study revealed that Cronbach's α for the SSRS was 0.865, and for the three subscales it was 0.884 (subjective support), 0.911(objective support), and 0.875 (the availability of support).

Data Analysis

EpiData version 3.1 was used to establish the study's database. We eliminated the questions with missing data or quality issues. To ensure accuracy, two trained personnel entered the data after all the surveys were completed. IBM SPSS Version 19.0 and Excel were used for the data analysis. The normal distributions of the continuous variables were verified using P–P plots and K–S tests. Descriptive statistics, including numbers (n), percentages (%), means, and standard deviations (SD) were calculated for the demographic variables. We used one-way analysis of variance (ANOVA) or independent sample t–tests to compare group differences on the measures of the continuous variables. The chi–square (χ^2) test was used to compare differences in the categorical variables. Pearson's correlations were used to examine correlations among the continuous variables. Hierarchical regression

For peer review only - http://bmjopen.bmj.com/site/about/guidelines.xhtml

analysis was used to examine the associations of the demographic characteristics and the scores on the SSRS, EPQ–RSC, and TCSQ with PTSD symptoms. Statistics, including F values, R^2, R^2–changes (ΔR^2), standardized regression coefficients (β), and P–values for each step in the regression model were reported. All the study variables were tested for multicolinearity. A P–value <0.05 was considered to be statistically significance.

STROBE Statement

We declared that the STROBE (Strengthening the Reporting of Observational Studies in Epidemiology) guidelines are followed in this study.

Ethical Considerations

Ethical approval to conduct this study was granted by the Research Ethics Committee of Harbin Medical University, and informed consent was obtained from each hospital and healthcare worker involved in the investigation. All of the participants gave their informed consent before the survey; they were assured that their personal information would be kept confidential.

RESULTS

Demographic Characteristics of the Respondents

The demographic characteristics of the respondents are shown in Table 1.

Table 1. Demographic characteristics of the respondents (N=2706).

Demographic variables		n	%
Gender	Male	623	23.0
	Female	2083	77.0
Age group	≤ 30	1258	46.5
	31–50	1341	49.5

For peer review only - http://bmjopen.bmj.com/site/about/guidelines.xhtml

	≥ 51	107	4.0
Educational level	Junior college or below	856	31.6
	Undergraduate	1341	49.6
	Graduate	509	18.8
Marital status	Married	1859	68.7
	Single/divorced/widowed	847	31.3
Occupation	Physician	1058	39.1
	Nurse	1520	56.2
	Medical Technician	128	4.7
Technical title	Primary	1147	42.4
	Intermediate	1026	37.9
	Senior	533	19.7
Department	Emergency Department	323	11.9
	Internal Medicine	813	30.0
	Surgery	752	27.8
	Obstetrics and Gynecology	276	10.2
	Pediatrics	218	8.1
	Other	324	12.0
Years of experience	≤ 4	1014	37.5
	5–10	820	30.3
	11–20	503	18.6
	≥ 21	369	13.6

Characteristics of the Victims in Relation to PTSD Symptoms

Of the 368 victims of physical violence, 59.8% were women, 51.3% completed an undergraduate education, and 73.9% were married. The characteristics of the victims in relation to PTSD symptoms are presented in Table 2.

Table 2. Characteristics of victims in relation to PTSD symptoms (N=368).

Variables		n	%	PTSD symptoms		F/t	*P*
				Mean	SD		
Gender	Male	148	40.2	44.03	16.19	3.537	0.000
	Female	220	59.8	38.30	13.71		
Age group	≤ 30	133	36.1	38.09	13.36	2.946	0.054
	31–50	216	58.7	42.01	15.56		
	≥ 51	19	5.2	42.10	17.80		
Educational level	Junior college or below	118	32.1	38.14	13.54	2.592	0.076
	Undergraduate	189	51.3	42.13	15.46		
	Graduate	61	16.6	40.64	15.85		
Marital status	Married	272	73.9	41.51	15.75	2.195	0.029
	Single/divorced/widowed	96	26.1	38.03	12.38		

For peer review only - http://bmjopen.bmj.com/site/about/guidelines.xhtml

For peer review only

Occupation	Physician	175	47.6	42.97	15.37	4.379	0.013
	Nurse	180	48.9	38.29	13.82		
	Medical Technician	13	3.5	40.69	21.24		
Technical title	Primary	145	39.4	39.56	13.04	0.576	0.562
	Intermediate	126	34.2	41.32	16.21		
	Senior	97	26.4	41.23	16.16		
Department	Emergency Department	68	18.5	41.46	16.08	0.722	0.607
	Internal Medicine	76	20.7	38.45	15.07		
	Surgery	123	33.4	41.53	13.91		
	Obstetrics and Gynecology	19	5.2	41.63	16.52		
	Pediatrics	27	7.3	37.63	10.37		
	Other	55	14.9	41.55	17.28		
Years of experience	≤ 4	101	27.4	37.19	13.25	2.158	0.063
	5–10	120	32.6	42.13	14.52		
	11–20	87	23.7	41.90	16.80		
	≥ 21	60	16.3	41.42	15.46		
Social support	Low	224	60.9	42.41	15.06	5.904	0.003
	Medium	130	35.3	38.52	14.40		
	High	14	3.8	31.00	14.53		
Subjective support	Low	22	6.0	41.91	18.63	0.859	0.425
	Medium	38	10.3	43.37	12.93		
	High	308	83.7	40.17	14.97		
Objective support	Low	206	56.0	42.19	15.47	3.369	0.035
	Medium	155	42.1	38.87	14.32		
	High	7	1.9	32.00	9.24		
Utilization of support	Low	39	10.6	48.18	16.98	6.979	0.001
	Medium	259	70.4	40.37	14.56		
	High	70	19.0	37.24	14.23		
Extraversion	Introversion	102	27.7	39.45	13.35	1.278	0.280
	Middle	164	44.6	41.99	16.04		
	Extraversion	102	27.7	39.51	14.80		
Psychoticism	Mild	68	18.5	42.22	16.87	0.998	0.370
	Middle	213	57.9	40.79	15.24		
	Tough-minded	87	23.6	38.86	12.69		
Neuroticism	Emotional instability	100	27.2	40.33	13.80	0.530	0.589
	Middle	153	41.6	41.50	16.72		
	Emotional stability	115	31.2	39.63	13.60		

Note. PTSD = post–traumatic stress disorder; SD = standard deviations.

Prevalence of Physical Violence in the Previous 12 Months

During the past 12 months, the prevalence of physical violence and psychological violence toward healthcare workers were 13.60% (368/2706) and 59.64% (1614/2706), respectively. The respondents

For peer review only

reported that the patients' relatives were the main perpetrators (67.4%, n = 248), followed by the patients (23.6%, n = 87).

Prevalence of PTSD

The PTSD symptoms based on the victims' PCL–C scores are summarized in Table 3. According to their scores on the PCL–C, 103 victims (28.0%) met the full criteria for a PTSD diagnosis and 21.2% of victims were at risk for developing PTSD.

According to the DSM IV–TR criteria for PTSD,[2] 47.0% of the victims did not appear to manifest the diagnostic criteria. Re–experiencing was the most frequently observed criterion for PTSD observed among the victims (45.1%), followed by hyper–arousal (37.8%).

Table 3. Sample description and prevalence of PTSD.

PTSD symptoms	Physical violence	
	n	%
**PTSD symptoms based on PCL–C scores**		
No obvious PTSD symptoms (17–37)	187	50.8
Criteria met for potential risk of PTSD (38–49)	78	21.2
Criteria met for the full PTSD diagnosis (50–85)	103	28.0
**PTSD symptoms based on PTSD criterion***		
No criterion manifestation	173	47.0
Re-experiencing (Criterion B)	166	45.1
Avoidance (Criterion C)	129	35.1
Hyper-arousal (Criterion D)	139	37.8

Note. PTSD = post–traumatic stress disorder; PCL–C = PTSD Checklist-Civilian Version.
*Participants may have more than one criteria.

Correlations of the EPQ–RSC, TCSQ and SSRS Scores with PTSD Symptoms

Table 4 shows the correlations among the victims' PTSD symptoms and scores on the EPQ–RSC, TCSQ, SRSS, and the physical violence subscale. The mean score for PTSD symptoms on the PCL–C was 40.60 (SD = 15.01).As expected, the level of PTSD symptoms was negatively correlated with respondents' scores on the SSRS (r=-0.188, P< 0.001) and positive coping subscale of the TCSQ (r = -0.164, P = 0.002), respectively. Physical violence was positively associated with PTSD symptoms

For peer review only - http://bmjopen.bmj.com/site/about/guidelines.xhtml

Table 4. Pearson correlations among PTSD symptoms, EPQ–RSC, TCSQ, SSRS and Physical Violence.

Variables	Mean	SD	1	2	3	4	5	6	7	8	9	10	11	12	13	14
1.PTSD symptoms	40.60	15.01	-													
2.Re-experiencing	12.43	4.92	0.89**	-												
3.Avoidance	15.70	5.99	0.94**	0.77**	-											
4.Hyper-arousal	12.46	5.48	0.91**	0.70**	0.79**	-										
5.Physical violence	3.08	2.99	0.26**	0.22**	0.31**	0.18**	-									
6.SSRS	41.73	8.44	-0.19**	-0.12**	-0.22**	-0.17**	-0.12**	-								
7.Subjective support	25.23	5.14	-0.09	-0.02	-0.13*	-0.09	0.01	0.89**	-							
8.Objective support	8.63	3.30	-0.21**	-0.17**	-0.23**	-0.17**	-0.20**	0.77**	0.46**	-						
9.Utilization of support	7.87	2.01	-0.21**	-0.15**	-0.22**	-0.20**	-0.21**	0.66**	0.43**	0.40**	-					
10.Positive coping of TCSQ	30.05	7.22	-0.16**	-0.15**	-0.12*	-0.18**	-0.13*	0.10	0.04	0.10*	0.15**	-				
11.Negative coping of TCSQ	26.92	7.33	0.19**	0.10	0.19**	0.22**	0.04	-0.31**	-0.26**	-0.29**	-0.17**	0.12*	-			
12.Extraversion	49.81	10.33	-0.01	-0.03	0.01	-0.01	0.06	-0.05	-0.02	-0.08	-0.02	-0.04	0.02	-		
13.Psychoticism	50.10	9.81	0.06	0.06	0.06	0.04	0.00	0.02	0.02	0.06	0.02	0.02	0.04	-0.02	-	
14.Neuroticism	50.07	10.34	0.03	0.04	0.01	0.05	0.03	0.03	0.01	0.05	0.04	0.01	0.04	-0.09	0.20**	-

Note. SD = standard deviations; PTSD = post–-traumatic stress disorder; EPQ–RSC = Eysenck Personality Questionnaire–Revised Short Scale for Chinese; SSRS = Social Support Rating Scale; TCSQ = Trait Coping Style Questionnaire.

$^{*}P<0.05$, $^{**}P<0.01$

For peer review only - http://bmjopen.bmj.com/site/about/guidelines.xhtml

(r = 0.259, P <0.001) .The level of PTSD symptoms was positively correlated with victims' scores on the negative coping subscale of the TCSQ (r = 0.188, P < 0.001).

Hierarchical Regression Analysis of Factors Related to PTSD Symptoms

The results of the hierarchical regression analysis are presented in Table 5.Variables that had a statistically significant association with PTSD were used as control variables. Gender had a significant effect on PTSD symptoms in the model (Block 1).As shown in Block 2, physical violence was positively associated with PTSD symptoms (β = 1.216, P < 0.001). As shown in Block 3, positive coping as measured by the TCSQ was negatively associated with PTSD symptoms (β = -0.327, P = 0.002), whereas, negative coping was positively associated with PTSD symptoms in the regression model(β = 0.353, P = 0.001). Furthermore, gender had a significant effect on PTSD symptoms, and men were more vulnerable to PTSD symptoms than women (Table 1). Therefore, we explored the potential correlates of PTSD symptoms in men and women (Table 6). As shown in Block 3,among the women, positive coping as measured by the TCSQ, was significantly associated with PTSD symptoms (β = -0.376, P = 0.001), but the effect of positive coping was not significant in men.

Table 5. Hierarchical regression for exploring the correlates of PTSD symptoms.

Variables	Block 1 (β)	Block 2 (β)	Block 3 (β)	Block 4 (β)
Gender	-4.663*	-3.282	-3.060	-3.012
Marital status	-2.021	-1.859	-2.626	-2.798
Occupation	-1.274	-1.918	-2.494	-2.414
Physical violence		1.216**	1.015**	1.028**
SSRS			-0.193*	-0.192*
Positive coping of TCSQ			-0.327**	-0.325**
Negative coping of TCSQ			0.353**	0.361**
Extraversion				-0.049
Psychoticism				0.081
Neuroticism				0.042
F	5.189**	9.886**	10.544**	11.584**
R^2	0.041	0.098	0.170	0.246
ΔR^2	0.041	0.057**	0.072**	0.076*

Note. PTSD = post–traumatic stress disorder; SSRS = Social Support Rating Scale; TCSQ = Trait

For peer review only - http://bmjopen.bmj.com/site/about/guidelines.xhtml

Coping Style Questionnaire.
*P<0.05, **P<0.01

Table 6. Hierarchical regression for exploring the correlates of PTSD symptoms in men and women, respectively.

	Variables	Mean (SD)	Block 1 (β)	Block 2 (β)	Block 3 (β)
Male					
n = 148	Physical violence	3.60 (3.16)	1.216**	1.033*	1.073**
	SSRS	41.25 (9.32)		-0.255	-0.257
	Positive coping of TCSQ	30.75 (7.28)		-0.298	-0.318
	Negative coping of TCSQ	27.21 (6.61)		0.467*	0.479*
	Extraversion	49.18 (10.04)			0.062
	Psychoticism	50.02 (8.62)			-0.282
	Neuroticism	49.99 (10.59)			-0.072
	F		8.727**	5.961**	4.182**
	R^2		0.056	0.143	0.173
	ΔR^2		0.056**	0.087**	0.030
Female					
n = 220	Physical violence	2.73 (2.83)	1.169**	0.955**	0.953**
	SSRS	42.05 (7.79)		-0.057	-0.066
	Positive coping of TCSQ	29.58 (7.16)		-0.376**	-0.376**
	Negative coping of TCSQ	26.72 (7.78)		0.296*	0.297*
	Extraversion	50.03 (10.54)			-0.121
	Psychoticism	50.14 (10.56)			0.006
	Neuroticism	50.12 (10.20)			-0.005
	F		13.441**	7.488**	4.557**
	R^2		0.058	0.122	0.131
	ΔR^2		0.058**	0.064**	0.009

Note. SD = standard deviations; PTSD = post–traumatic stress disorder; SSRS = Social Support Rating Scale; TCSQ = Trait Coping Style Questionnaire.
*P<0.05, **P<0.01

DISCUSSION

In this cross–sectional hospital–based study of healthcare workers exposed to physical violence, we assessed the prevalence and correlates of PTSD symptoms. Our study found that the prevalence of physical violence among the healthcare workers was approximately 13.6% in the previous year. The results of a study conducted during 2009–2010 in Italy found that 13.4% of nurses were exposed to physical violence,[22] which is similar to the frequency found in this study. However, other studies have

For peer review only - http://bmjopen.bmj.com/site/about/guidelines.xhtml

reported higher prevalence rates of physical violence than the current study.[23-24] The inconsistency in these findings may be attributed to cultural differences between countries or missing reports. PTSD was reported by 28.0% of the victims based on the scoring instructions of the PCL–C (i.e., 28.0% scored 50 points and above). We selected the PCL–C score of 50 and above as the standard cut–off due to the influence of traditional Chinese culture on the frequency of healthcare workers' encounters with traumatic events and the DSM IV–TR criteria for PTSD.[2] Previous studies have provided valuable information regarding the prevalence of PTSD among doctors and nurses.[28-31]The prevalence of PTSD among the healthcare workers exposed physical violence in our study was similar to that reported in Atlanta.[55] However, the prevalence rates of PTSD in these studies were different from the present study,[56-57] which might be attributed to differences in the studies' sample characteristics, designs, definitions, and diagnostic criteria for PTSD, due to their varied cultural backgrounds. Moreover, the prevalence of PTSD symptoms in our sample was higher than that of the general population (8%) in the USA.[58]This finding might be attributed to the fact that the general population's frequency of exposure to serious traumatic events is lower than that of healthcare workers. Similarly, nurses who work in intensive care units experience traumatic events more often than other healthcare workers do.[29]

Our study found that 21.2% of the victims of physical violence were at risk for developing PTSD and 28.0% met the full diagnostic criteria for PTSD. This finding suggests that physical violence had a strong influence on the mental health of healthcare workers. Approximately 53.0% (195/368) of the victims reported having at least one PTSD criterion. The most commonly observed PTSD symptom was re–experiencing (45.1%), followed by hyper–arousal (37.8%), and then avoidance (35.1%). A previous study also reported that healthcare workers in an emergency department were victims of direct workplace violence because they reported re–experiencing the violent event, followed by

For peer review only - http://bmjopen.bmj.com/site/about/guidelines.xhtml

hyper–arousal and avoidance.[28] Laposa and Alden reported that re–experiencing an incident of physical violence was significantly and negatively associated with emergency department workers' ability to accomplish their work.[28] It is possible that the prevalence of symptoms of hyper–arousal and avoidance were not higher due to the distinctive characteristics of the healthcare workers' jobs and the hospital's culture, which required them to be able to shift their focus quickly and constantly. Healthcare workers who escaped slight injury during an episode of physical violence had to shift their focus rapidly to another patient after the event, and they could not avoid the work environment.[28]

As shown in the results of the Pearson's correlations and the hierarchical regression analysis, social support had a significant negative association with PTSD symptoms, and this finding is consistent with other research.[9 36 52 53] The level of PTSD symptoms was significantly and negatively correlated with the healthcare workers' scores for objective support and utilization of support. A previous study found that the Deterioration Model of Social Support has been useful in discriminating the potential of stressors to reduce support.[58]They found that disaster–induced erosion of perceived social support increased symptoms of depression among both primary and secondary victims; the loss of perceived social support also mediated psychological consequences.[59] The Deterioration Deterrence Model of Social Support, which is similar to support–mobilization models, has been used to explain how the perceived deterioration of social support can be counteracted by higher levels of received social support.[59-60] If post–disaster support mobilization is implemented, stress should be positively correlated with received support. At the same time, received support should be positively related to perceived support. Therefore, the receipt of support should suppress a negative relationship between stress and perceived support.[59-60] Victims of physical violence should be encouraged not to abandon their daily social activities because these activities have many important functions (e.g., they help

For peer review only - http://bmjopen.bmj.com/site/about/guidelines.xhtml

people understand the needs of network members and inspire their participation in helping).[60] Daily contact is the most natural forum for sharing experiences, which might suppress negative emotions, provide opportunities for social comparison, and maintain a sense of friendship and feelings of being accepted.[60] It is important to recognize that stress caused by violence is persistent. Yet, a supportive hospital environment can help individuals cope with a wide range of stressful events and serve as a buffer against their negative health effects.[59-60]

Another significant effect of coping styles on PTSD symptoms was found in the present study. This result indicated that when healthcare workers encountered a traumatic event, a negative coping style was more likely to increase their proneness to developing PTSD symptoms. This finding is consistent with the results of other studies.[36 50 53] Positive coping was beneficial in preventing or alleviating PTSD symptoms in our study. In contrast, a previous investigation found that activing coping had a positive relationship with PTSD.[36] Unexpectedly, the three personality factors were not significantly associated with PTSD symptoms. However, Lawrence and Fauerbach's study found that individuals with higher Neuroticism scores exhibited more PTSD symptoms.[36]

An important finding of the present study was revealed in the univariate analyses. We found that the men exposed to traumatic events were more likely to exhibit PTSD symptoms than the women were. This result was different from the findings reported in earlier studies that women are more likely to develop PTSD symptoms.[6 17 19] This finding might be attributed to gender differences in responses to different traumatic events and in social networks.[61-62] This phenomenon also might be attributable to the fact that the injuries sustained by the men after experiencing physical violence were more severe than those of the women. After experiencing physical violence, the women were likely to receive more social support than the men suggesting that women were more often regarded as a vulnerable group.

For peer review only - http://bmjopen.bmj.com/site/about/guidelines.xhtml

These findings suggest that social support, exposure to physical violence, and coping styles are closely related to PTSD symptoms. Therefore, we recommend interventions based on the social cognitive theory.[63] For example, social support has been found to be an important protective factor in reducing stress and depression, and improving health.[63] After the occurrence of a traumatic event, enabling function of social support can enhance self-efficacy, thereby promoting recovery from the trauma.[63]

The present study has several limitations. First, we used the PCL-C to assess PTSD symptoms rather than a standard clinical diagnostic method. Consequently, the prevalence of PTSD might be overestimated. Second, the study's findings need to be replicated in a longitudinal study. Finally, convenience sampling is a non-probability sampling method and the results of this study are specific to Chinese healthcare workers exposed to physical violence in the past 12-months.Thus, the low representativeness of the sample due to the use convenience sampling limits the generalizability of the results. The inclusion of healthcare workers from a wider range of careers in a more representative sample should contribute to the ability to generalize the results of future studies.

CONCLUSIONS

The results suggest that the aftermath of physical violence contributes to the current prevalence of PTSD. The positive effects of social support on PTSD symptoms suggest that social support has practical implications for psychological interventions to promote mental health. Furthermore, the coping styles of the healthcare workers in this study influenced the development of PTSD symptoms. Therefore, it is imperative to use positive coping methods and to receive social support after experiencing a traumatic event.

For peer review only - http://bmjopen.bmj.com/site/about/guidelines.xhtml

Acknowledgments

The authors thank all the healthcare workers, managers and Chinese Hospital Association for their assistance and support for this project.

Author Contributions

LS and LF designed the study. LS, LW, XJ, BP and LF collected data. ZL, LW, XJ, HM, XL and AL analysed the data. LS and LF drafted the manuscript. LS, ZL and LF revised the manuscript.

Funding

This study was funded by the National Natural Science Foundation of China, grant number 71473063.

Competing interests None declared.

Data sharing statement No additional data are available.

REFERENCES

1. Guo W, Xue JM, Shao D, *et al.* Effect of the interplay between trauma severity and trait neuroticism on posttraumatic stress disorder symptoms among adolescents exposed to a pipeline explosion. *PLoS One* 2015;10(3):e0120493.
2. Association AP. Diagnostic and statistical manual of mental disorders fourth edition, text revision (DSM-IV-TR). 2000.
3. Andreasen NC. What is post-traumatic stress disorder? *Dialogues Clin Neurosci* 2011;13:240–243.
4. Association AP. Diagnostic and statistical manual of mental disorders fifth edition, text revision (DSM-V). 2013.
5. Van Der, Kolk BA.The psychobiology and psychopharmacology of PTSD. *Hum Psychopharmacol* 2001;16(S1):S49 – S64.

For peer review only - http://bmjopen.bmj.com/site/about/guidelines.xhtml

6. Shalev AY. Post-traumatic stress disorder. Disorder takes away human dignity and character. *BMJ* 2001;322(7297):1301–1304.

7. Hu BS, Liang YX. Post-traumatic stress disorder. *J Occup Environ Hyg* 1997;(5):266-269.

8. Klein S, Alexander DA. Epidemiology and presentation of post-traumatic disorders. *Psychiatry* 2009;8(8):282-287.

9. Fan F, Zhang Y, Yang Y, *et al.* Symptoms of posttraumatic stress disorder, depression, and anxiety among adolescents following the 2008 Wenchuan earthquake in China. *J Trauma Stress* 2011;24(1): 44–53.

10. Boe HJ, Holgersen KH, Holen A. Mental health outcomes and predictors of chronic disorders after the North Sea oil rig disaster: 27-year longitudinal follow-up study. *J Nerv Ment Dis* 2011;199(1):49–54.

11. Wang YL, Xie W, Yang ZH, *et al.* Research progress of posttraumatic stress disorder in China. *Chinese Journal of Applied Psychology* 2005;11(2):176-180.

12. Alexander DA, Klein S. Ambulance personnel and critical incidents: impact of accident and emergency work on mental health and emotional well-being. *Brit J Psychia* 2001;178(1):76.

13. Jonsson A, Segesten K, Mattsson B. Post-traumatic stress among Swedish ambulance personnel. *Emerg Med J* 2003;20(1):79-84.

14. Laposa JM, Alden LE, Fullerton LM. Work stress and posttraumatic stress disorder in ED nurses /personnel. *J Emerg Nurs* 2003;29(1):23-28.

15. Shoji K, Lesnierowska M, Smoktunowicz E, *et al*. What Comes First, Job Burnout or Secondary Traumatic Stress? Findings from Two Longitudinal Studies from the U.S. and Poland. *Plos One* 2015;10(8):e0136730.

For peer review only - http://bmjopen.bmj.com/site/about/guidelines.xhtml

16. Boer JD, Lok A, Verlaat EV, *et al*. Work-related critical incidents in hospital-based health care providers and the risk of post-traumatic stress symptoms, anxiety, and depression: A meta-ana lysis. *Soc Sci Med* 2011;73(2):316-326.

17. Krug EG, Mercy JA, Dahlberg LL, *et al*. The world report on violence and health. *Lancet* 2002; 360(9339):1083.

18. Ray MM. The dark side of the job: Violence in the emergency department. *J Emerg Nurs* 2007;33 :257-261.

19. Ahmad M, Al-Rimawi R, Masadeh A, *et al.* Workplace Violence by Patients and their Families against Nurses: Literature Review. *Int J NursTermin Cl* 2015;2(4):46-55.

20. Ramacciati N, Ceccagnoli A, Addey B. Violence against nurses in the triage area: An Italian qualitative study. *Int Emerg Nurs* 2015;23(4):274-280.

21. Harrell, Erika. Workplace Violence, 1993-2009: National Crime Victimization Survey and the Census of Fatal Occupational Injuries. *Digitalcommons* 2011.

22. Magnavita N, Heponiemi T. Workplace violence against nursing students and nurses: an Italian experience. *J Nurs Scholarsh* 2011;43(2):203-210.

23. Fute M, Mengesha ZB, Wakgari N, *et al*. High prevalence of workplace violence among nurses working at public health facilities in Southern Ethiopia.*BMC Nurs* 2015;14:9.

24. Al-Omari H. Physical and verbal workplace violence against nurses in Jordan. *Int Nurs Rev* 2015;62(1):111–118.

25. Schablon A, Zeh A, Wendeler D, *et al.* Frequency and consequences of violence and aggression towards employees in the German healthcare and welfare system: a cross-sectional study. *BMJ Open* 2011;2(5):421-421.

For peer review only - http://bmjopen.bmj.com/site/about/guidelines.xhtml

26. Najafi F, Fallahi-Khoshknab M, Dalvandi A, *et al.* Workplace violence against Iranian nurses: A systematic review. *J Health Promot Manage* 2014;3:72-85.

27. Pan Y, Yang XH, He JP, *et al*. To be or not to be a doctor, that is the question: a review of serious incidents of violence against doctors in China from 2003–2013. *J public health* 2015;23(2):111 -116

28. Laposa JM, Alden LE. Posttraumatic stress disorder in the emergency room: exploration of a cognitive model. *Behav Res Ther* 2003;41(1):49-65.

29. Mills LD, Mills TJ. Symptoms of post-traumatic stress disorder among emergency medicine residents. *J Emerg Med* 2005;28(1):1–4.

30. Friedman, MJ, Resick, PA, Bryant, R.A, *et al.* Considering PTSD for DSM-5. *Depress Anxiety* 2011;28(9):750-769.

31. Robertson N, Perry A. Institutionally based health care workers' exposure to traumatogenic events: Systematic review of PTSD presentation. *Journal of Traumatic Stress* 2010;23(3):417-420.

32. Kornblith AB, Herndon JE, Weiss RB, *et al.* Long-term adjustment of survivors of early-stage breast carcinoma, 20 years after adjuvant chemotherapy.*Cancer*2003;98(4):679–689.

33. Rourke MT, Hobbie WL, Schwartz L, *et al.* Posttrauamatic stress disorder (PTSD) in young adult survivors of childhood cancer. *Pediatr Blood Cancer* 2007; 49(2): 177–82.

34. Perrin M, Vandeleur C L, Castelao E, *et al*. Determinants of the development of post-traumatic stress disorder, in the general population. *Soc Psych Psych Epid* 2014;49(3):447-457.

35. Breslau N, Davis G C, Andreski P, *et al*. Traumatic events and posttraumatic stress disorder in an urban population of young adults. *Arch Gen Psychiatry* 1991;48(3):216-222.

For peer review only - http://bmjopen.bmj.com/site/about/guidelines.xhtml

36. Lawrence JW, Fauerbach JA. Personality, coping, chronic stress, social support and PTSD symptoms among adult burn survivors: a path analysis. *J Burn Care Rehabil* 2003;24(1):63.

37. Kerasiotis B, Motta RW. Assessment of PTSD symptoms in emergency room, intensive care unit, and general floor nurses. *Int J Emerg Ment Health* 2004;6(3):121-133.

38. Boer JD, Lok A, Verlaat EV, *et al*. Work-related critical incidents in hospital-based health care providers and the risk of post-traumatic stress symptoms, anxiety, and depression: A meta-analys is. *Soc Scie Med* 2011;73(2):316-326.

39. Shoji K, Lesnierowska M, Smoktunowicz E, *et al*. What Comes First, Job Burnout or Secondary Traumatic Stress? Findings from Two Longitudinal Studies from the U.S. and Poland. *Plos One* 2015;10(8):e0136730.

40. Cieslak R, Anderson V, Bock J, *et al*. Secondary traumatic stress among mental health providers working with the military: Prevalence and its work- and exposure-related correlates. *J Nerv Ment Dis* 2013;201(11):917-25.

41. ILO/ICN/WHO/PSI. Framework Guidelines for Addressing Workplace Violence in the Health Sector. Geneva: ILO/ICN/WHO/PSI Joint Programme on Workplace Violence in the Health Sector; 2002.

42. Zampieron A, Galeazzo M, Turra S, *et al.* Perceived aggression towards nurses: study in two Italian health institutions. *J Clin Nurs* 2010;19:2329–41.

43. Battles ED. An exploration of post-traumatic stress disorder in emergency nurses following Hurricane Katrina. *J Emerg Nurs* 2007;33(4):314-318.

44. Dirk Richter K B. Post-traumatic stress disorder following patient assaults among staff members of mental health hospitals: a prospective longitudinal study. *BMC Psychiatry* 2006;6(1):15.

For peer review only - http://bmjopen.bmj.com/site/about/guidelines.xhtml

45. Weathers FW, Litz BT, Herman DS, *et al*. The PTSD checklist: reliability, validity & diagnostic utility. Paper presented at the Annual Meeting of the International Society for Traumatic Stress Studies, *San Antonio, TX* 1993.

46. Wu Z, Xu J, Sui Y. Posttraumatic stress disorder and posttraumatic growth coexistence and the risk factors in Wenchuan earthquake survivors. *Psychiatry Res* 2016; 237:49-54.

47. Qian M, Wu G, Zhu R, *et al.* Development of the revised Eysenck Personality Questionnaire Short Scale for Chinese (EPQ-RSC). *Acta Psychologica Sinica* 2000;32(3):317-323.

48. Zhang Z, Shi Z, Wang L, *et al.* Post-traumatic stress disorder, anxiety and depression among the elderly: a survey of the hard-hit areas a year after the Wenchuan earthquake. *Stress Health* 2012; 28(1):61-68.

49. Cui Y, Tian SS, Qiao N, *et al.* Associations of Individual-Related and Job-Related Risk Factors with Nonfatal Occupational Injury in the Coal Workers of Shanxi Province: A Cross-Sectional Study. *PLoS One* 2015;10(7):e0134367.

50. Zhang L, Zhang J X, Wen-Juan X U, *et al.* An evaluation of validity and reliability on the trait coping style questionnaire(TCSQ) using for Chinese soldier. *Chinese Journal of Disease Control & Prevention* 2010.

51. Li-Na L I, Zhao Y, Cui X J, et al. The Influence Factors and Status on Male Vicarious Trauma of Disaster. *Chinese General Practice* 2014.

52. Feng S, Tan H, Benjamin A, *et al.* Social support and posttraumatic stress disorder among flood victims in Hunan, China. *Ann Epidemio*l. 2007;17(10):827-833.

53. Li H, Yan C, Zhu S, *et al.* Correlation among coping style, social support, and negative emotion in infertile women. Zhong nan da xue xue bao. Yi xue ban=Journal of Central South University.

For peer review only - http://bmjopen.bmj.com/site/about/guidelines.xhtml

Medical sciences 2011;36(2):138-142.

54. Chang-Fei LU, Jia CX, Zhang JY, *et al.* Reliability and validity of Social Support Rating Scale in rural suicides. *Chinese Mental Health Journal* 2011;25(3):218-222.

55. Mealer ML, Shelton A, Berg B, *et al*. Increased prevalence of post-traumatic stress disorder symptoms in critical care nurses. *Am J Resp Crit Care* 2007;175(175):693-697.

56. Zafar W, Khan UR, Siddiqui SA, *et al*. Workplace Violence and Self-reported Psychological Health: Coping with Post-traumatic Stress, Mental Distress, and Burnout among Physicians Working in the Emergency Departments Compared to Other Specialties in Pakistan. *J Emerg Med* 2015;50(1):167-177.

57. Johansen VA, Eilertsen DE, Nordanger D, *et al.* Prevalence, comorbidity and stability of post-traumatic stress disorder and anxiety and depression symptoms after exposure to physical assault: an 8-year prospective longitudinal study. *Nord J Psychiatry* 2013; 67(1):69-80.

58. Breslau N. The epidemiology of post-traumatic stress disorder; what is the extent of the problem? *Journal of Clinical Psychiatric* 2001;62:16-22.

59. Kaniasty K, Norris FH. Mobilization and deterioration of social support following natural disasters. *Current Directions in Psychological Science* 1995;4(3):94-98.

60. Norris FH, Kaniasty K. Received and perceived social support in times of stress: a test of the social support deterioration deterrence model. *Journal of Personality & Social Psychology* 1996;71(3):498-511.

61. Freedman SA, Gluck N, Tuval-Mashiach R, *et al*. Gender differences in responses to traumatic events: a prospective study. *J Trauma Stress* 2002;15:407-413.

62. Pratchett LC, Pelcovitz MR, Yehuda R. Trauma and violence: are women the weaker sex?

For peer review only - http://bmjopen.bmj.com/site/about/guidelines.xhtml

Psychiatr Clin North Am 2010; 33: 465–474.

63. Benight CC, Bandura A. Social cognitive theory of posttraumatic recovery: the role of perceived self-efficacy. *Behaviour Research & Therapy* 2004;42(10):1129.

For peer review only

For peer review only - http://bmjopen.bmj.com/site/about/guidelines.xhtml

STROBE 2007 (v4) Statement—Checklist of items that should be included in reports of *cross-sectional studies*

Section/Topic	Item #	Recommendation	Reported on page #
**Title and abstract**	1	(*a*) Indicate the study's design with a commonly used term in the title or the abstract	Page 1, line 6; p. 2 , line 21
		(*b*) Provide in the abstract an informative and balanced summary of what was done and what was found	P. 2 ; p.3, line 6-17
**Introduction**			
Background/rationale	2	Explain the scientific background and rationale for the investigation being reported	P. 4; p. 5; p. 6, line 4-32
Objectives	3	State specific objectives, including any prespecified hypotheses	P. 6, line 34-39
**Methods**			
Study design	4	Present key elements of study design early in the paper	P. 7, line 14-24
Setting	5	Describe the setting, locations, and relevant dates, including periods of recruitment, exposure, follow-up, and data collection	P. 6, line 47-58; p. 7, line 4-14; line 24-39
Participants	6	(*a*) Give the eligibility criteria, and the sources and methods of selection of participants	P. 7, line 42-57; p.8, 4
Variables	7	Clearly define all outcomes, exposures, predictors, potential confounders, and effect modifiers. Give diagnostic criteria, if applicable	P. 8, line 11-29
Data sources/	8*	For each variable of interest, give sources of data and details of methods of assessment (measurement). Describe	P. 8, line 31-57; p.9;

For peer review only

measurement		comparability of assessment methods if there is more than one group	p.10; p. 11, line 4-39
Bias	9	Describe any efforts to address potential sources of bias	P. 6, line 57; p. 7,line 4
Study size	10	Explain how the study size was arrived at	P. 7, line 24-32
Quantitative variables	11	Explain how quantitative variables were handled in the analyses. If applicable, describe which groupings were chosen and why	p. 11, line 52-17; p. 12, line 4-6; P.6, line 11-31
Statistical methods	12	(*a*) Describe all statistical methods, including those used to control for confounding	P. 11, line 52-57; p. 12, line 2-24
		(*b*) Describe any methods used to examine subgroups and interactions	P. 12, line 4-16
		(*c*) Explain how missing data were addressed	P. 11, line 47-52
		(*d*) If applicable, describe analytical methods taking account of sampling strategy	P. 12, line 4-16
		(*e*) Describe any sensitivity analyses	P. 11, line 54; p. 12, line 16-24
**Results**			
Participants	13*	(a) Report numbers of individuals at each stage of study—eg numbers potentially eligible, examined for eligibility, confirmed eligible, included in the study, completing follow-up, and analysed	P. 13, line 42-47
		(b) Give reasons for non-participation at each stage	P. 7, line 47-57
		(c) Consider use of a flow diagram	No
Descriptive data	14*	(a) Give characteristics of study participants (eg demographic, clinical, social) and information on exposures and potential confounders	P. 13; p.14

For peer review only

		(b) Indicate number of participants with missing data for each variable of interest	P. 11, line 47-49
Outcome data	15*	Report numbers of outcome events or summary measures	P. 15, line 4-50
Main results	16	(*a*) Give unadjusted estimates and, if applicable, confounder-adjusted estimates and their precision (eg, 95% confidence interval). Make clear which confounders were adjusted for and why they were included	P. 17, line 17-58; p. 18, line 3-47
		(*b*) Report category boundaries when continuous variables were categorized	P. 8, line 9-29
		(*c*) If relevant, consider translating estimates of relative risk into absolute risk for a meaningful time period	P. 13, line42-59; p. 14; p.16
Other analyses	17	Report other analyses done—eg analyses of subgroups and interactions, and sensitivity analyses	P. 17, line 16-44
**Discussion**			
Key results	18	Summarise key results with reference to study objectives	P. 18, line 54-57; p. 19. Line 4-9
Limitations	19	Discuss limitations of the study, taking into account sources of potential bias or imprecision. Discuss both direction and magnitude of any potential bias	P. 22, Line 27-42
Interpretation	20	Give a cautious overall interpretation of results considering objectives, limitations, multiplicity of analyses, results from similar studies, and other relevant evidence	P. 19; p.20; p.21; p. 22, line4-42
Generalisability	21	Discuss the generalisability (external validity) of the study results	P. 22, line 12-42
**Other information**			
Funding	22	Give the source of funding and the role of the funders for the present study and, if applicable, for the original study on which the present article is based	P. 23, line 27-29

*Give information separately for cases and controls in case-control studies and, if applicable, for exposed and unexposed groups in cohort and cross-sectional studies.

For peer review only

Note: An Explanation and Elaboration article discusses each checklist item and gives methodological background and published examples of transparent reporting. The STROBE checklist is best used in conjunction with this article (freely available on the Web sites of PLoS Medicine at http://www.plosmedicine.org/, Annals of Internal Medicine at http://www.annals.org/, and Epidemiology at http://www.epidem.com/). Information on the STROBE Initiative is available at www.strobe-statement.org.

For peer review only
